# Lipid Nanoparticles as a Promising Drug Delivery Carrier for Topical Ocular Therapy—An Overview on Recent Advances

**DOI:** 10.3390/pharmaceutics14030533

**Published:** 2022-02-27

**Authors:** Shery Jacob, Anroop B. Nair, Jigar Shah, Sumeet Gupta, Sai H. S. Boddu, Nagaraja Sreeharsha, Alex Joseph, Pottathil Shinu, Mohamed A. Morsy

**Affiliations:** 1 Department of Pharmaceutical Sciences, College of Pharmacy, Gulf Medical University, Ajman 4184, United Arab Emirates; 2 Department of Pharmaceutical Sciences, College of Clinical Pharmacy, King Faisal University, Al-Ahsa 31982, Saudi Arabia; anair@kfu.edu.sa (A.B.N.); sharsha@kfu.edu.sa (N.S.); momorsy@kfu.edu.sa (M.A.M.); 3 Department of Pharmaceutics, Institute of Pharmacy, Nirma University, Ahmedabad 382481, India; jigsh12@gmail.com; 4 Department of Pharmacology, M. M. College of Pharmacy, Maharishi Markandeshwar (Deemed to be University), Mullana 133203, India; sumeetgupta25@gmail.com; 5 Department of Pharmaceutical Sciences, College of Pharmacy and Health Sciences, Ajman University, Ajman 346, United Arab Emirates; s.boddu@ajman.ac.ae; 6 Department of Pharmaceutics, Vidya Siri College of Pharmacy, Off Sarjapura Road, Bangalore 560035, India; 7 Department of Pharmaceutical Chemistry, Manipal College of Pharmaceutical Sciences, Manipal Academy of Higher Education, Manipal 576104, India; alex.joseph@manipal.edu; 8 Department of Biomedical Sciences, College of Clinical Pharmacy, King Faisal University, Al-Ahsa 31982, Saudi Arabia; spottathail@kfu.edu.sa; 9 Department of Pharmacology, Faculty of Medicine, Minia University, El-Minia 61511, Egypt

**Keywords:** lipid nanoparticles, ocular drug delivery, solid-lipid nanoparticles, nanostructured lipid carriers, nanoemulsions, liposomes, clinical trials

## Abstract

Due to complicated anatomical and physical properties, targeted drug delivery to ocular tissues continues to be a key challenge for formulation scientists. Various attempts are currently being made to improve the in vivo performance of therapeutic molecules by encapsulating them in various nanocarrier systems or devices and administering them via invasive/non-invasive or minimally invasive drug administration methods. Biocompatible and biodegradable lipid nanoparticles have emerged as a potential alternative to conventional ocular drug delivery systems to overcome various ocular barriers. Lipid-based nanocarrier systems led to major technological advancements and therapeutic advantages during the last few decades of ocular therapy, such as high precorneal residence time, sustained drug release profile, minimum dosing frequency, decreased drug toxicity, targeted site delivery, and, therefore, an improvement in ocular bioavailability. In addition, such formulations can be given as fine dispersion in patient-friendly droppable preparation without causing blurred vision and ocular sensitivity reactions. The unique advantages of lipid nanoparticles, namely, solid lipid nanoparticles, nanostructured lipid carriers, nanoemulsions, and liposomes in intraocular targeted administration of various therapeutic drugs are extensively discussed. Ongoing and completed clinical trials of various liposome-based formulations and various characterization techniques designed for nanoemulsion in ocular delivery are tabulated. This review also describes diverse solid lipid nanoparticle preparation methods, procedures, advantages, and limitations. Functionalization approaches to overcome the drawbacks of lipid nanoparticles, as well as the exploration of new functional additives with the potential to improve the penetration of macromolecular pharmaceuticals, would quickly progress the challenging field of ocular drug delivery systems.

## 1. Introduction

The complex anatomy, physiology, and biochemistry of the human eye make it nearly inaccessible to foreign particulates, including drugs. As a result, developing an ocular drug delivery system remains a fascinating and difficult issue facing formulation and development experts. The key objective behind the design and development of an ocular drug delivery system is to offset the protective barriers of the eye to provide high therapeutic efficacy without inducing permanent tissue damage. However, the performance of many ophthalmic preparations is often restricted by short retention time, restricted permeability of corneal epithelium, high pre-corneal clearance rate due to rapid blinking rates (6–15 times/min), high tear turn over (0.5–2.2 μL/min), nasolacrimal discharge and non-productive conjunctival uptake [1,2]. Furthermore, the low retention volume (~30 μL) of the conjunctival sac typically results in decreased corneal or scleral transport of drugs [3]. The current review intends to summarize the recent progress and ocular drug delivery strategies involving lipid nanocarriers. The article also aims to discuss the emerging role of these nanosystems in treating both anterior and posterior segments of ocular diseases. Comprehensive knowledge of anatomical and physiological barriers of the ocular region, biochemical pathways in the ocular tissues, and drug transfer mechanisms via ocular epithelial surface are a prerequisite for the development of efficient ocular delivery systems.

## 2. Anatomical and Physiological Features of the Human Eye

**Figure 1:**
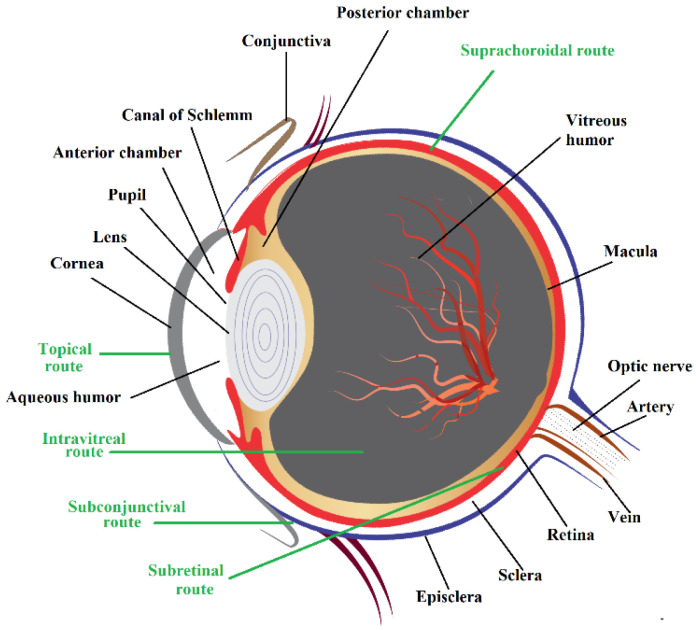
Schematic diagram depicting the key regions and various ocular routes of drug administration in the human eye.

The aqueous humor, cornea, conjunctiva, iris, ciliary body, and lens are all found in the anterior chamber of the human eye, whereas the vitreous humor, retina, sclera, choroid, and optic nerve are all found in the posterior chamber [4]. The cornea is a trilayered structure comprising of diffusional barriers namely, epithelium, stroma, and endothelium. The outermost 6–7-layered epithelium and an innermost simple squamous sheet of endothelial layer contain 100-fold more lipid material than intermediate stroma or substantia propria. The stroma is mainly composed of dense, regularly packed collagen fibrils organized in orthogonal layers or lamellar fashion. The basement membrane, or Descemet’s membrane, is a comparatively transparent and collagen-rich matrix that is present between the stroma and the endothelial layer of the cornea. Bowman’s membrane lies just beneath the epithelium, which is mostly made up of collagen fibers, an important protein that structurally reinforces the cornea. Distal to Bowman’s membrane, narrow, flattened epithelial cells form zonula occludens or tight interjunctional complexes. The aqueous humor present in anterior as well as posterior segments maintains the intraocular pressure, while the gelatinous and transparent vitreous humor secreted by the ciliary body fills the vitreous chamber [5]. Schematic representation displaying key regions and various ocular routes of the human eye is depicted in Figure 1.

## 3. Ocular Drug Delivery Barriers

**Figure 2:**
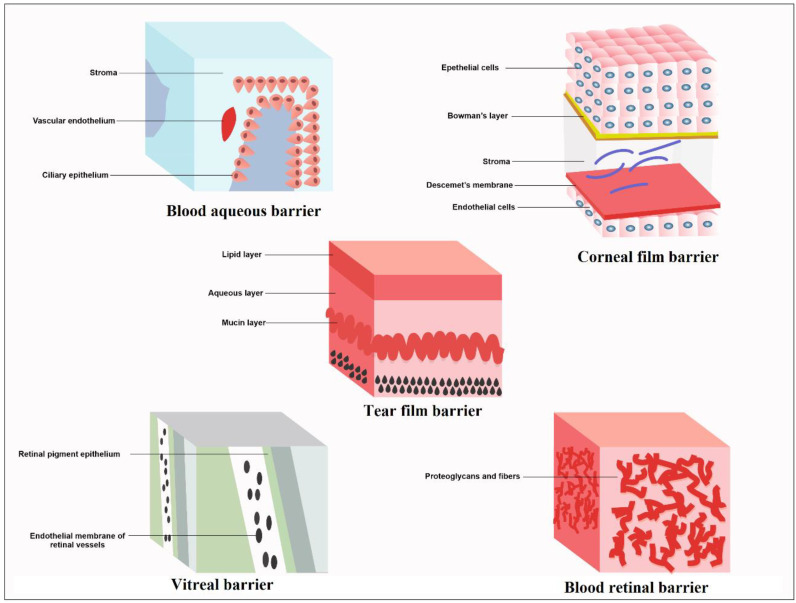
Schematic diagram depicting various ocular barriers for drug intake in the human eye.

Schematic representation of various ocular barriers to drug absorption in the human eye is depicted in Figure 2. Drug absorption is hampered by the corneal and conjunctival epithelial cells that cover the ocular surface. The existence of the blood–ocular barrier restricts the hydrophilic macromolecules from entering the systemic circulation and also avoids the re-entry of xenobiotics from the systemic circulation towards the eye. The anterior blood-aqueous barrier and the posterior blood–retinal barrier make up the blood-ocular barrier system [6]. The blood-aqueous humor consists of non-fenestrated vascular endothelial cells covering the iris blood vessels and tight junctions (zonula occludens) connecting the apical portions of adjacent epithelial cells of the non-pigmented ciliary body epithelium [7]. The inner retinal microvascular endothelium forms the blood–retinal barrier, while the outer retinal–blood barrier is generated by the retinal pigment epithelial cells [8]. The tight and restrictive physiological barrier of blood and retina maintains retinal homeostasis by regulating the transport of nutrients, electrolytes, proteins, peptides, and osmotic water flows into and out of the retina. It’s worth noting that the most common posterior segment retinal illnesses, such as diabetic retinopathy and age-related macular degeneration, are linked to changes in the blood–retinal barrier. The aqueous tear film layer acts as a barrier between corneal surface and external environment. It is typically comprised of innermost mucin, lipocalin and lysozyme-enriched aqueous layer and outermost lipid layer. It was disclosed that specific blood-vitreal barriers do exists based on the varying concentrations of diverse molecules found in the vitreous and aqueous. Glaucoma, allergic conjunctivitis, anterior uveitis, and cataracts are among common diseases that affect the anterior segment.

## 4. Drug Transport Mechanisms

Topical delivery of the drug into cul-de-sac may be absorbed through either a non-corneal route by diffusion across sclera and conjunctiva or via corneal membrane into the intraocular tissues. Due to certain beneficial properties, such as avoidance of systemic absorption, as well as hepatic metabolism and ease of drug administration, the topical route is preferred to treat superficial infections and inflammation, besides conditions such as glaucoma or uveitis that affect the anterior segment of the eye [9,10]. It has been stated that the ocular bioavailability from topical solution is only 5–10% due to reduced entry of drugs to inner ocular tissues; it is difficult to attain targeted drug concentration, particularly into the posterior segment [11].

**Figure 3:**
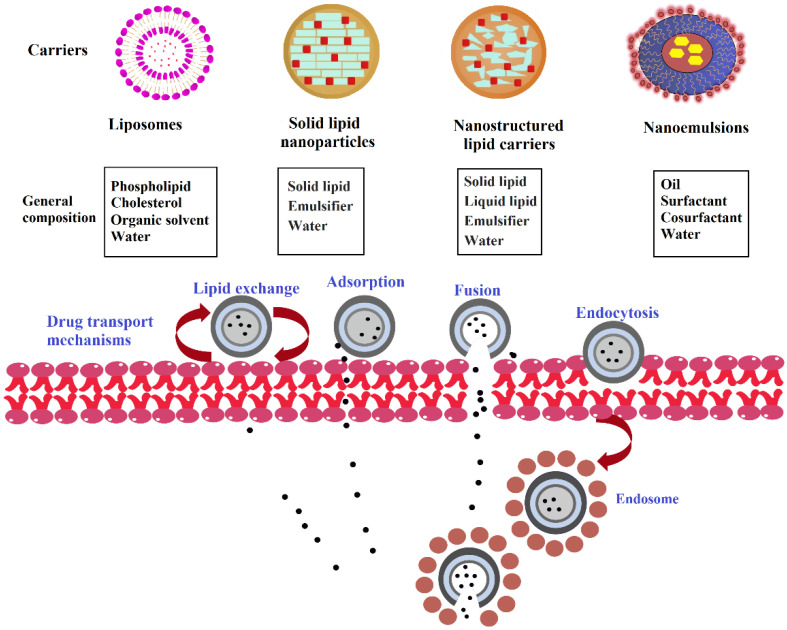
Schematic diagram depicting various drug transport mechanisms of lipid nanoparticles in the human eye.

In comparison to the non-corneal route of absorption, transcellular transport through the corneal epithelial membrane and stroma represents the major mechanism of absorption for most therapeutic agents. The corneal permeability depends on physicochemical properties of actives such as surface area, diffusivity, concentration gradient, pKa, optimum log *p* value between 1–3, which is also comparable with other biological membranes [3,12]. Though the corneal membrane has tight interjunctional complexes, the presence of an intracellular pore size of nearly 60 Å permits small ionic and hydrophilic drugs to gain access to the intraocular tissue through the paracellular pathway. The corneal epithelial cells tend to remain as a depot for drugs and to release them into the corneal stroma, which is extremely hydrophilic. This would allow for the permeation of polar drugs with molecular weight up to 50 kDa but exert a rate-limiting membrane barrier to highly hydrophobic drugs [13]. Hence, the molecular size, solubility, lipophilicity, and degree of ionization will have a significant influence on drug penetration and ocular bioavailability. The iris-ciliary body expresses the number of drug transporters belonging to the ATP-binding cassette family [14], various multidrug resistance-associated proteins, and solute carrier families [15]. Investigations revealed that drug transporter exists in the iris-ciliary body hinders the diffusion of drugs from blood-to aqueous humor and active clearance of the drug from the aqueous humor resulting in low ocular bioavailability. Alteration of the blood-aqueous barrier due to various ocular conditions, such as inflammation, intraocular surgery, trauma, or vascular diseases, can disrupt the membrane integrity and homeostasis of the eye [16]. Static (corneal membrane and anterior blood-aqueous barrier), dynamic (conjunctival blood flow, lymphatic drainage, and tear turnover), and metabolic barriers all limit medication delivery to the anterior portion of the eye [17]. Lipid transfer or exchange to the cellular or subcellular membrane, fusion with the plasma membrane, adsorption to the ocular surface through weak hydrophobic or electrostatic forces, and endocytosis by phagocytic cells of the reticuloendothelial cells can be considered as probable drug transportation mechanisms of lipid nanoparticles (Figure 3).

The intracameral injection represents drug administration directly to the anterior chamber of the eye, but prior administration of general anesthesia and potential damage to intraocular structures often restricts this mode of delivery [18]. The intravitreal injection can deliver small molecules (<500 Da) directly to the vitreous humor, but repeated administration of drugs through this route can cause complications linked to the retina and increased intraocular pressure [19]. Though highly invasive techniques such as subretinal injection are useful for gene delivery [20], they can lead to frequent complications, such as vitreous hemorrhage, retinal detachment, recurrence of submacular hemorrhage, intraocular pressure, and post-operative development of choroidal neovascularization [21]. Drugs are frequently administered through periocular routes by subconjunctival, intrascleral, sub-Tenon’s retrobulbar, and peribulbar injections to deliver them directly to posterior ocular tissues, thus, avoiding the risks of endophthalmitis and retinal damage [22]. Various dosage forms, such as eye drops, hydrogels, in situ gels, nanoparticles, nano micelles, polymeric ocular inserts, implants, dendrimers, nanosuspensions, and microneedles, have been developed to improve ocular bioavailability by prolonging the precorneal residence time and corneal penetration of the applied drugs [23,24,25,26,27].

## 5. Nanocarriers in Ocular Drug Delivery

Due to submicron particle size and peculiar physicochemical characteristics possessed by the nanocarriers, they can act as an efficient delivery vehicle to transport actives to site-specific targets. Nanocarriers are typically constituted of particulate, soluble, or are attached with target-specific ligands for various drug delivery applications [28]. They are capable of encapsulating different types of active (s) formulated from diverse materials by utilizing various preparation techniques. Nanoparticles are extensively explored to develop novel drug delivery systems capable of facilitating actives to penetrate through various physiological barriers that exist in the ocular region [23]. They can also act as a reservoir or depot to release the drug slowly after endocytosis by the epithelial cells of the cornea. By providing sustained release of drugs, these nanocarriers prevent rapid loss of drug via nasolacrimal discharge and rapid tear turnover. In addition, inhibition of p-glycoprotein activity [29] present in epithelial cells and opening up tight junctions of the cornea by non-ionic surface active agents of the formulation can likely improve the ocular bioavailability [30]. When treating illnesses of the posterior segment of the eye, they can operate as a controlled release mechanism, avoiding the need for repeated drug administration. Biodegradable and biocompatible polymers are used to fabricate nanoparticles in which the drug is either dissolved, dispersed, and/or surface-bound [31].

In this context, various efforts are being attempted to improve the pre-corneal contact time and trans-corneal permeability properties that could potentially enhance intraocular bioavailability [32]. In recent years, colloidal nanoparticulate lipid systems viz. solid lipid nanoparticles (SLN) [33], nanostructured lipid carriers (NLC) [34], nanoemulsion [35], and liposomes [36] have gained wide attention as a favorable drug delivery vehicle in both anterior and posterior ocular diseases. Numerous benefits are associated with lipid nanoparticles, such as modified release, improved uptake, high stability, low degradability, in vivo compatibility, and adaptability to various delivery routes [1,37]. Biocompatible and biodegradable lipids utilized to prepare these nanosystems have the significant ability to reduce the adverse effects of the ophthalmic preparations [38]. Compared to polymeric nanoparticles, numerous benefits are associated with lipid nanoparticles, such as modified release, excellent stability, minimum decomposition of lipids, in vivo tolerability, and adaptability to various delivery routes, which enables lipid nanoparticles as a suitable and efficient drug transporting vehicle in different delivery systems [39]. Moreover, lipid nanoparticles can encapsulate hydrophobic and hydrophilic drugs, improve the bioavailability of low water-soluble actives, and protect them from premature elimination. The lipid materials typically employed to develop these nanocarriers are non-toxic, non-immunogenic and, therefore, exhibit remarkable tissue compatibility, and tolerability properties. The key distinctive feature between NLCs and SLNs is the nature of lipids included in the formulation, fluid lipids in NLCs, and solid lipids in SLNs. Over the last few decades, nanoemulsions were extensively evaluated as a delivery vehicle for hydrophobic drugs [40]. Nevertheless, the practicability of modified drug release from nanoemulsions is rather limited because of the nanosized particles and the liquid state of the nanosystems.

### 5.1. Solid-Lipid Nanoparticles

SLN emerged as a dominant lipid-based nanocarrier in diverse drug delivery systems. The nano-sized (10–1000 nm) particles of SLN are conventionally prepared by dispersing a solid lipid matrix in an aqueous phase comprised of surfactant as stabilizing agent [41]. SLNs have shown numerous benefits over other colloidal carriers, such as modified drug release, site-specific drug delivery, long-term stability, high entrapment efficiency, biocompatibility, sterilizable, formulated as self-administrable eye drops, simple production steps, and ease of scale-up [42]. SLNs can, additionally, protect the sensitive lipophilic drugs from degradation because of the immobile state of these agents in the solid-state of lipid matrix compared to the fluid phase [43]. These nanocarriers have shown tremendous potential to be administered through parenteral, peroral, transdermal, pulmonary, nasal, ocular, rectal, and vaginal routes [44,45,46].

SLNs have the potential ability for rapid diffusion across the corneal membrane and are largely distributed in ocular structures. Furthermore, enhanced interaction and adhesion between SLNs and the corneal endothelial membrane barrier would allow them to be considered as an attractive delivery tool for ocular drug transport [47]. Due to the possession of various desirable characteristics, SLNs are incorporated as an efficient drug carrier in the ophthalmic drug delivery system to prolong ocular residence time, enhance corneal absorption, improve ocular bioavailability, and offer sustained drug release [48]. The main disadvantages associated with SLNs are lipid particle growth, aggregation, solidification, polymorphic transition and low drug loading ability because of the crystalline nature of solid-lipid [49]. Another major limitation of SLN is the initial burst effect contributed by the adsorbed drug on the peripheral surface of nanoparticles, particularly observed with hydrophilic drugs. Because of the crystalline solid lipid core of SLNs, the polar drugs are mainly located in the outer surfactant layer; therefore, loading capacity is rather limited. To improve the drug loading, as well as to minimize the leakage of hydrophilic drugs, the lipid drug conjugates chemically linked to a lipoidal molecule, such as fatty acids or phospholipids, have been developed [50]. The release behavior of SLNs is mainly influenced by the location of the drug, whether it is on the surface of lipid matrices, differences in the drug deposition within the particle, or the polymorphic transition of the solid lipid matrix.

#### 5.1.1. Components of Solid Lipid Nanoparticles

The main ingredients typically used in the preparation of SLNs are solid lipid, emulsifier, and water. The most commonly used solid lipids as a structural component of SLN are triglycerides (tristearin), partial glycerides (glyceryl monostearate), fatty acids (stearic acid), fatty acid esters (glyceryl behenate), steroids (cholesterol), and waxes (cetyl palmitate) [51]. To reduce the mean particle size of the lipid formulations, a combination of long and short-chain fatty acids is typically used. Most of these lipids are generally regarded as safe and are approved by European Union and US regulatory authorities.

Stabilizing agents such as surfactants are included to lower the interfacial tension formed at the boundary between the lipid and the aqueous phase of the SLN formulation [52]. They tend to adsorb as a flexible and mechanically strong monolayer at the interface and, thus, to impart physical stability to the nanodispersion during manufacturing and storage. Important factors that should be considered for the selection of surfactant(s) are hydrophilic–lipophilic balance, biodegradability, cytocompatibility, impact on the lipid crystallinity/polymorphism, particle size, etc. [53]. The surfactants typically used in the preparation are non-ionic namely, Tween 20, Tween 60, Tween 80, poloxamer 182, poloxamer 188, poloxamer 407, tyloxapol; the negatively charged surfactants such as sodium lauryl sulfate, sodium cholate, and sodium glycolate; the cationic surfactants viz. 1,2-dioleoyl-3-trimethylammonium-propane and cetyltrimethylammonium bromide (CTAB), in addition to the amphoteric surfactants, are biological membrane lipids such as soybean lecithin and egg lecithin [46,48,54]. Frequently used co-surfactants in the preparation of SLNs are polyvinyl alcohol (PVA), butanol, propylene glycol, and polyethylene glycol (PEG). Cationic surfactants such as 1,2-dioleoyl-3-trimethylammonium and CTAB may be utilized to enhance corneal drug penetration because of ionic interactions with anionic epithelial cells [46].

One of the important parameters influencing the adequate dispersibility of drugs in the lipid matrix in SLNs and NLCs is significant lipophilicity (log P > 2) [55]. In general, potential drug candidates recommended for lipid formulations are neutral or basic with low melting temperature (<150 °C), polar functional groups, and adequate solubility of the drug in lipids and water [56]. The development of lipid nanocarriers encapsulated with hydrophilic drugs might face formulation issues, such as low entrapment efficiency and limited stability. In such cases, partitioning of the drug towards the external phase may adversely affect mucoadhesiveness, cell uptake, and desired drug release essential for ophthalmic formulations. For instance, lipid nanoparticles prepared from different fluoroquinolone derivatives demonstrated maximum encapsulation efficiency for low aqueous soluble ofloxacin (20%) followed by levofloxacin and lowest (4%) for maximum water-soluble, ciprofloxacin [57]. Additional ingredients include cryoprotectants, namely, glucose, sorbitol, and fructose in lyophilized SLN formulation, coating polymer such as chitosan antimicrobial preservative, e.g., para-aminobenzoic acid esters, organic mercurial compounds, benzyl alcohol, potassium sorbate, phenoxyethanol and tocopherol [58]. Frequently used structural components of solid-lipid nanoparticles are summarized in Table 1.

#### 5.1.2. Preparation Methods

The preparation techniques of SLN are mainly based on the utilization of either high energy, low energy, or organic solvents. The most extensively used high energy processes are high shear homogenization and/or ultrasonication, microwave-assisted, high-pressure homogenization, and hot and cold homogenization techniques. An overview of various SLN preparation methods, procedures, advantages, and limitations are given in Table 2. High shear homogenization is usually proceeded by ultra-sonication, which reduces the size of globules depending on the generation, nucleation, growth, and implosive rupture of bubbles [62]. The high energy method includes procedures such as supercritical fluid extraction of emulsion particles from gas saturated solution and gas-assisted melting atomization. In the case of the supercritical fluid extraction method, an oil-in-water (o/w) emulsion is initially prepared using either water-miscible or water-immiscible solvents and, subsequently, the organic solvent is extracted using suitable supercritical fluid. Generally, supercritical carbon dioxide is used, which quickly extracts the solvent leads to the precipitation of uniform-sized lipid nanoparticles [63]. In supercritical assisted injection in a liquid antisolvent technique, expansion of water-miscible organic solvent comprised of drugs, lipids, and supercritical carbon dioxide takes place when it is continuously injected into an aqueous medium comprising surfactant (antisolvent). Blending these two fluids leads to spontaneous supersaturation and rapid precipitation of lipid nanoparticles [64].

A novel method for the treatment of diabetic retinopathy with SLNs containing siRNA to silence HuR expression has been described [74]. It was demonstrated that the animals treated with coated siRNA demonstrated significant retinal protection via reduction of HuR and VEGF compared to naked siRNA. When administered through contact lenses, PEGylated SLNs loaded with latanoprost were found to decreases the intraocular pressure by raising the increasing uveoscleral outflow [75]. Ocular gene therapy incorporating genetically engineered non-viral vectors to express a particular protein sequence for treating different retinal genetic diseases, namely, retinitis pigmentosa, Stargardt disease, Leber congenital amaurosis, or X-linked juvenile retinoschisis has been demonstrated in various clinical trials [76,77].

A successful RS1 gene transfer to Rs1h-deficient animals using SLN embedded with non-viral vectors such as hyaluronic acid or dextran has shown promising results for the treatment of X-linked juvenile retinoschisis [78]. Fifteen days after subretinal or intravitreal injection to *Rs1h*-deficient mice, green fluorescent protein and retinoschisin expression were observed in all retinal layers indicating a partial recovery of the retina. SLNs have been also used as nonviral vector carriers for cell-specific gene delivery employing retinoschisin specific photoreceptors, murine opsin promoters. It was found that hyaluronic acid-SLN resulted in a significantly higher increase in the thickness of both retina and outer nuclear layer, which can be interpreted as the higher transfection capacity of murine opsin promoter [79].

In the coacervation technique, a mixture comprising salts of fatty acids and aqueous phase constituting polymeric stabilizing agent is heated to Kraft temperature point until a transparent alkaline micellar salt solution of lipid is obtained [80]. To allow the precipitation of SLNs, an acidic or coacervating solution is introduced dropwise to the above solution and cooled. The drug is usually dissolved in alcohol and later incorporated in the lipid phase or added to the blank SLNs [81]. Different types of low energy approaches are viz. micro emulsion-based, membrane contractor technique, phase inversion temperature, coacervation, and double emulsion techniques [39]. Typical SLN preparation methods utilizing organic solvents are solvent emulsification/evaporation, solvent emulsification-diffusion, solvent injection techniques. Furthermore, SLNs can be developed employing hot-melt extrusion and cross-shaped microchannel methods as well.

In vitro degradation and in vivo toxicity studies have been carried out on various lipids typically used to fabricate SLNs. The literature suggests that SLNs comprised of cetyl palmitate are well tolerated for parenteral administration though it is not a physiological compound [82]. In vitro studies in human plasma showed that the use of an extremely high dose (~100 g) of Compritol^®^ is limited by side effects due to slow metabolic degradation. Nevertheless, SLN prepared from Compritol^®^ are recognized as being appropriate for intravenous use since the administered dose is very less during therapy. It is worthwhile to note that most of the lipids at a concentration utilized for the fabrication of SLN are physiologically compatible and biodegradable. Toxicity evaluation of risperidone SLN formulations using Caco-2 cells by (4,5-dimthylthiazol-2-yl)2,5-diphenyl-tetrazolium bromide assay disclosed that all formulations are biocompatible and well-tolerated [83].

#### 5.1.3. In Vitro Characterization Techniques for Solid-Lipid Nanoparticles

During the last few decades, great advancements have been made in various techniques utilized for in vitro characterization of SLNs. The lipids in SLNs can undergo crystallization tendencies and polymorphic changes during formulation and storage and can affect the system’s stability. Therefore, the characterization of lipids in SLN formulation is significant to compare compared to other lipid-based nanoparticles. Particle size is a critical evaluation parameter that is typically determined using dynamic light scattering or photon correlation spectroscopy, the laser diffraction technique, coulter counting, scanning ion occlusion sensing, and flow field fractionation methods [84]. Shape and surface features have a great impact on the metabolic fate and performance of the nanoparticles. High-resolution qualitative analytical techniques such as scanning electron microscopy and transmission electron microscopy are routinely used to find the particle shape and size morphology. However, the electron beam utilized in these methods can melt the lipids, thereby, affecting the structural integrity of the nanoparticles. This limitation can be avoided by using an enhanced imaging technique known as Cryo-field emission scanning electron microscopy [84]. Freeze drying employed in this technique would prevent the collapse of the SLN structure during analysis. The surface charge or zeta potential of the particles can be measured using electrophoretic light scattering and electroacoustic techniques [85]. The determination of polymorphism and lipid crystallization is routinely carried out using differential scanning calorimeter and X-ray diffraction. The temperature must be suitably controlled during thermal scanning to avoid the decomposition of lipids. Evaluation of critical formulation parameters such as entrapment efficiency and drug loading is important to determine the efficiency of the prepared SLNs. Ultra-centrifugation, gel-exclusion chromatography, and ultra-filtration techniques are extensively used to separate nanoparticles from dispersion medium and subsequent analysis using various analytical techniques [86]. The dissolution or release studies can be carried out in a dialysis bag apparatus using a suitable buffer medium and maintenance of sink condition at a specific temperature.

#### 5.1.4. Functional Role of Solid-Lipid Nanoparticles in Ocular Delivery

The main objective of the SLN in the ocular drug delivery is to extend the retention time of applied formulation with the ocular epithelium, thereby enhancing corneal permeation via various transport mechanisms. Further, SLN dispersed in mucoadhesive polymeric gel formulation has the additional benefits of controlling the drug release and extended stability. Interpenetration and entanglement of polymer chains with mucin are responsible for mucoadhesion to ocular epithelia. Polymer hydration, swelling, and mucin dehydration are the main mechanisms underlying mucoadhesive strength.

Mucins have many critical functional roles in the ocular tissues, such as hydration, lubrication, and management of tear flow to facilitate smooth blinking, act as protective cell surface barrier, trap and eliminate allergens, pathogens, and debris; they also aid in the diffusion of essential nutrients and oxygen [87]. Two major mucins are expressed by the ocular surface epithelium: cell surface-associated mucins MUC1, -4, and -16, and the gel-forming mucin MUC5AC, which is released by the conjunctival goblet cells. The carboxyl and sulfate groups present in the oligosaccharide chains confer a negative charge to the mucins. The functional role of SLN in ocular drug delivery of various therapeutic categories, their typical characteristics, and important highlights are depicted in Table 3.

It was reported that spatial charge distribution within mucin matrices have a critical role in selective mucosal transport, design, and development of drug delivery carrier with modifiable transport characteristics [93]. The capacity of chitosan, a biocompatible, cationic mucoadhesive polymer, to electrostatically interact with the negatively charged sulfate and sialic acid residues present in mucin’s oligosaccharide chain, has been extensively researched [94]. Mucoadhesive ability can be further enhanced by functional group alteration through chemical modification of the existing polymers.

Cationic lipid nanoparticles are presumed to enhance ocular bioavailability because they enable electrostatic interactions with the anionic ophthalmic mucosal surface resulting in prolonged retention time of the drug. Epigallocatechin gallate embedded positively charged SLNs (EGCG-SLNs) were prepared by multiple emulsion techniques using various cationic surface-active agents such as CTAB and dimethyl dioctadecyl ammonium bromide (DDAB). Drug-loaded SLNs were evaluated for modified release and site delivery properties [95]. Dynamic laser diffraction studies demonstrated nanosized (<150 nm) particles of EGCG-SLNs and a polydispersity index value around 0.25. In vitro drug release study in the simulated physiological buffer at 37 °C indicated faster release (>50% in 4 h) from solution, when compared with the EGCG-SLNs. The results from trans-corneal and transscleral permeation studies disclosed that corneal permeability and steady-state flux of lipophilic EGCG-CTAB nanoparticles were 3-times higher than EGCG dimethyl dioctadecyl ammonium bromide (EGCGDDAB) nanocarriers. In contrast, hydrophilic EGCG-DDAB particles showed a 3-fold enhancement of transscleral permeation compared to EGCG-CTAB particles. The investigation also confirmed the constant permeation rate of EGCG via ocular structures and extended-release profile up to 6 h. The in vitro hen’s egg test chorioallantoic membrane (HET-CAM) and in vivo Draize test studies confirmed that the developed formulations are non-toxic and non-irritant.

Functionalized chitosan-based SLNs were also used for enhanced corneal permeation and efficient ocular delivery [96]. A modified emulsion-solvent evaporation approach, for example, was used to create methazolamide-loaded SLNs made of low molecular weight chitosan. The particle size (199.4 ± 2.8 nm and 252.8 ± 4.0 nm) and zeta potential (−21.3 ± 1.9 mV and +31.3 ± 1.7 mV) of plain SLNs loaded with methazolamide and cationic chitosan SLNs with methazolamide were found significant. Extended in vitro release patterns and enhanced ex vivo permeation through rabbit cornea were demonstrated in chitosan-SLNs compared to SLNs loaded with methazolamide. In addition, in vivo studies displayed the significant intraocular pressure-lowering effect of chitosan SLNs (245.75 ± 18.31 mmHg/h) in comparison to both plain SLNs (126.74 ± 17.73 mmHg/h) and marketed ophthalmic drops (171.17 ± 16.45 mmHg/h). Moreover, the physically stable chitosan SLNs did not show any ocular irritancy based on the Draize method and histological examination.

The thiolated conjugate of cysteine-PEG monostearate was utilized for fabricating NLCs loaded with cyclosporin A for ocular delivery [97]. The in vitro release of cyclosporin A release from lipid nanoparticles was slower compared to non-thiolated counterparts because of extensive cross-linking between thiomers and ocular epithelia. In vivo evaluation in rabbits demonstrated that cyclosporine A level in systemic circulation was near to the sensitivity level. These data revealed that the thiolated NLC can transfer a greater quantity of cyclosporine A to deeper intraocular structures because of its inherent mucoadhesive nature and sustained release property. Due to significant physicochemical stability, SLNs can be incorporated in thermoresponsive gel to extend the duration of contact with the cornea and prevent premature precorneal elimination due to nasolacrimal discharge [98].

**Figure 4:**
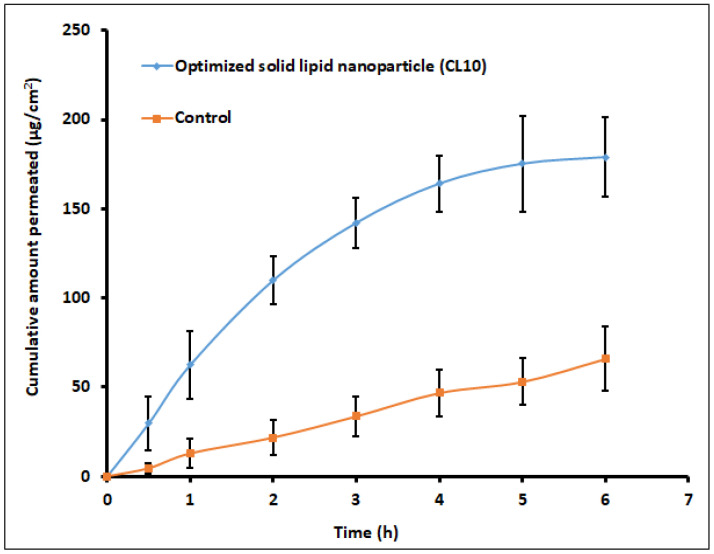
The total amount of clarithromycin that passed through the goat cornea as a result of the optimized solid lipid nanoparticles formulation and the control (solution) constituted an equivalent dose (adapted from [1], published by MDPI, 2021).

In a recent investigation, our research group formulated SLNs to increase the trans-corneal transport and evaluate ocular pharmacokinetics of clarithromycin in the rabbit model [1]. High-speed stirring and ultrasonication were used to make SLNs with stearic acid as a lipid former, tween 80 as a surfactant, and transcutol P as a cosurfactant. The in vitro release profile of optimized SLNs (CL10) demonstrated ~80% drug release in 8 h and higher ex vivo transport (30.45 µg/cm^2^/h; *p* < 0.0001) through goat corneal membrane as compared to control (10.94 µg/cm^2^/h). Pharmacokinetic evaluation of selected formulation (CL10) in New Zealand Albino rabbits indicated significant enhancement of clarithromycin bioavailability (*p* < 0.0001) confirmed based on a 150% increase of C_max_ (~1066 ng/mL) and a 2.8-fold rise in AUC in comparison to control solution. In distinct bacterial endophthalmitis, the mean drug concentration observed in the aqueous humor (Figure 4) was greater than the minimum inhibitory concentration of clarithromycin. Being a non-invasive approach, topical drug delivery utilizing SLNs could potentially improve therapeutic outcomes in the treatment of various infections caused in the anterior segment of the eye and, thus, enhance patient compliance.

Diseases that involve the posterior chamber of the eye are now recognized as the leading cause of visual impairment globally [99]. Present therapy demands non-invasive methods resulting in serious adverse events, in addition to multiple administrations [100]. Lipid-based nanosystems illustrate promising results, in addition to being more safe and efficient in treating posterior segment diseases.

Pores on the ocular structures assist the intracellular transport of SLN and NLC particles with a size range of 200–300 nm. Furthermore, nanoparticles with nearly 100 nm are engulfed by active receptor-mediated phagocytosis that existed in cells in both the corneal and conjunctival pathways. These internalization mechanisms confirmed by in vitro, in vivo, and ex vivo methods suggest a significant benefit of lipid systems in ocular drug transport [42,89]. Retinal drug delivery utilizing SLNs as nonviral vectors for gene therapy has been reported [47,79]. The use of SLN integrated with non-viral vectors such as hyaluronic acid or dextran to successfully transfer the RS1 gene to Rs1h-deficient animals has shown encouraging results for the treatment of X-linked retinoschisis [78].

### 5.2. Nanostructured Lipid Carriers

NLCs are a delivery system that uses an emulsifying agent to disperse partially-crystallized colloidal lipid particles with an average particle size ≤100 nm in an aqueous phase. According to one theory, the included fluid oil can exist as small globules that solubilize a significant portion of the medication and are stabilized by the surrounding solid lipid matrix, resulting in the formation of a new amorphous matrix with improved polymorphism behavior [101].

Lipid nanoparticles such as NLCs can solve the formulation challenges typically linked to the development of polymeric nanoparticles such as cytotoxicity, utilization of organic solvents, and difficulty to scale up for large-scale manufacturing [102]. Similar to SLNs, NLCs have extended retention time at the targeted ocular site, thereby improving the therapeutic efficacy while decreasing side-effects mainly contributed by their mucoadhesive property. Dynamic nanocarrier systems such as SLNs and NLCs are in a thermodynamically unstable state. This would allow high entrapment ability with improved mobility of the entrapped drugs. However, the transformation of lipid structure to a stable state causes the displacement of drug molecules during storage. It has been hypothesized that the release rate from SLNs is much slower compared to NLCs at low drug encapsulation while no significant differences in release rate were observed at high drug loading. The storage at room temperature reported that NLCs are comparatively more stable than SLNs [103]. NLCs have been studied extensively for the therapy of diverse ocular conditions, such as infections, inflammation, glaucoma, and disorders affecting the posterior segment of the eye and are summarized in Table 4.

Depending on the preparation process and lipid constitution of the matrix, there are imperfect, amorphous, and multiple types of NLC. The NLCs possess better characteristics as a drug delivery system overcoming typical formulation constraints associated with SLNs, such as high lipid crystallinity and improved long-term stability. Further, combining solid and lipid matrix in NLCs leads to less ordered lipid matrix structure with enhanced drug entrapment and minimum drug expulsion during storage. Imparting mucoadhesive to nanocarrier by providing surface retentive properties could potentially increase their precorneal contact time and ocular bioavailability. An improved mucoadhesion has been demonstrated with an NLC surface coated with cationic, chitosan oligosaccharide designed for ocular drug delivery applications [111]. The surface coating over NLC was confirmed with surface analysis techniques such as small-angle neuron scattering and X-ray photoelectron spectroscopy. The chitosan-coated NLC was found to remain on the ocular surface more than the uncoated NLC during the 4 h study. Furthermore, a higher concentration of the loaded drug, etoposide, was estimated compared to the uncoated NLC. Increased etoposide concentration might be due to the penetration-enhancing ability of chitosan at the corneal epithelial surface or by reversibly affecting various ocular transportation pathways without having any adverse effects on cell viability [112]. This study indicates that, to achieve the desired concentration of actives within the eye, adequate retention on the ocular surface is essential besides sufficient permeation. An increase in residence time and enhanced corneal penetration was demonstrated by formulating brimonidine in NLCs [113]. Drug-loaded NLCs were prepared by modified high shear homogenization using glyceryl monostearate poloxamer^®^ P 188 and castor oil. Formed NLCs were a spherical shape, exhibited negative zeta potential, high percentage entrapment efficiency, and low crystallinity index. The permeability coefficient of NLCs was 1.3 fold higher than that of SLN; the highest reduction (−13.14  ±  1.28 mmHg) of intraocular pressure was demonstrated with NLCs in rabbits.

Recently, a smart drug delivery system created from a nanohybrid system that combines the beneficial properties of each material was described. NLC can be immobilized in a hydrogel matrix covalently or noncovalently with adequate crosslinking density to prevent the untimely release of nanoparticles. In NLC-based hydrogel, it was reported that rehydration and re-dissolution of hydrogel films could lead to the recovery of NLC. Surprisingly, the structure and size of nanoparticles were restored even after reconstitution due to hysteresis. NLC-based hydrogel is expected to release the drug slowly since the drug must cross an additional barrier due to encapsulation within nanoparticles. In vitro study performed on dexamethasone-NLC hybrid hydrogel provide a cumulative drug release of 88.65% demonstrating sustained release up to 72 h while dexamethasone loaded in NLC showed a faster drug release profile with 93.10% of total dexamethasone delivered within 48 h [114]. The study confirms that NLC incorporated in hydrogel can act as an efficient nanocarrier for ocular sustained drug release. NLC incorporated in hydrogel can increase viscosity and, hence, the retention at the ocular site for an extended duration. NLC loaded with quercetin was formulated using melt-emulsification method followed by ultra-sonication technique [109]. The optimized quercetin NLC exhibited a particle size of 75.54 nm with homogenous size distribution and high entrapment efficiency (97.14%). It was dispersed and cross-linked in a pH and temperature dual-responsive hydrogel constituted of carboxymethyl chitosan and poloxamer 407 with a natural cross-linker, genipin. In vitro release studies indicated dual responsiveness of the hydrogel and 80.52% of total quercetin released in 72 h, demonstrating the sustainability of the nanohybrid hydrogel system. In summary, NLC-based hydrogel with suitable crosslinking ability can be considered as a potential and promising ophthalmic drug delivery system.

#### Preparation Methods

Different formulation techniques typically utilized for the preparation of NLCs are closely similar to SLNs, such as high-pressure homogenization, solvent emulsification-evaporation, phase inversion, high-speed homogenization, and/or ultrasonication, and solvent injection [115]. High-pressure homogenization is a simple and inexpensive method but has certain limitations, such as long exposure of the drug to high temperatures. The scale-up process is feasible with both solvent emulsification-evaporation and solvent injection/displacement method; however, use of organic solvent is a major disadvantage. Different temperature cycles required in the phase inversion technique make this preparation process more complex. High-speed homogenization and/or ultrasonication typically results in decreased particle size but suffers from possible contamination of the formulation with metal particles.

### 5.3. Nanoemulsions

Nanoemulsions are thermodynamically unstable, kinetically stable, optically clear, or translucent submicron (20–200 nm) isotropic colloidal dispersion system typically comprised of an aqueous and oil phase, surfactant as a primary emulsifying agent, intermediate-length alkanols as an auxiliary emulsifying agent, and, infrequently, an electrolyte [116]. It can be further classified into o/w, w/o, and bicontinuous types, based on the type and solubility characteristics of emulsifying agents based on Bancroft’s rule [117]. The leading advantages of this colloidal drug carrier include increased ocular residence and contact time, decreased drug-protein binding, rapid permeation across the barriers, sustained release, reduced systemic toxicity, and the benefit of incorporating both polar and nonpolar drugs. Nanoemulsions can additionally prevent the susceptible drug from undergoing hydrolysis and enzymatic degradation [118]. Moreover, nanoemulsions can adhere closely to the outermost tear film lipid layer of the conjunctival sac for a prolonged duration and, hence, serve as a drug depot [119]. It can be considered a viable substitute for standard ophthalmic dosage forms in treating many eye disorders that affect both the anterior and posterior ocular segments and is elaborated elsewhere due to its multiple benefits [120].

Typically, in situ nanoemulsions are positively charged and are preferred particularly for lipophilic drugs targeted against various ocular bacterial, fungal, viral infections, dry eye disease, and immune-mediated inflammatory anterior ocular disease [35,121,122]. Nanoemulsions improve corneal residence time and enhance permeation across the corneal tight junction, thereby enhancing the ocular bioavailability. This was endorsed by the FDA approval (2002) of Restasis^®^ (Allergan) and Cationorm^®^ (Novagali Pharma) by the European Union (2008) for the treatment of dry eye. Recently, cyclosporin A-based nanoemulsion, Ikervis^®,^ was approved for treating severe keratitis [122].

#### 5.3.1. Preparation Methods

Nanoemulsions are typically prepared by either energy-intensive processes namely, ultrasonication, high-pressure homogenization, high-shear mixing, microfluidic and membrane methods, or low energy methods such as phase inversion emulsification techniques [123]. In high-pressure homogenization, coarse emulsion at high pressure (500–5000 psi) is allowed to pass through the narrow aperture to generate nanoemulsion having globules size up to 1 nm [124]. Uniform-sized nanoemulsions are formed due to the generation of external forces, such as hydraulic shear, severe turbulence, and cavitation in the system. Although, applied over a short duration, high energy, and elevated temperature may degrade thermosensitive compounds such as proteins, peptides, and enzymes [125]. High stirring techniques utilize high-energy mixtures such as Silverson high shear mixers and high-speed rotor-stator systems for preparing a nanoemulsion. High-speed stirring leads to strong centrifugal force resulting in intense dispersion of emulsion [126].

Nanoemulsion can also be prepared by mixing an organic phase containing the dissolved drug, surfactant, and cosurfactant and then injecting it into a continuously stirred aqueous medium. Though this method is feasible for encapsulating thermolabile actives, the lack of emulsion stability limits the favorable outcome [127]. The ultrasound emulsification technique involves the creation of acoustic cavitation forces due to acoustic waves, which causes the generation and collapse of microbubbles. Furthermore, the formation of localized turbulence generating microimplosions and shock wave emissions eventually lead to the breakage of macro droplets to the nanosized emulsion. For the maximum efficiency and uniform particle size distribution, the emulsion must be recirculated several cycles to allow the maximum shear rate to all droplets. Denaturation of proteins, depolymerization of polymers, and oxidation of lipids are some of the problems typically associated with this method [128]. Microfluidizer provides high pressure to continuously force the coarse emulsion to an interaction chamber, wherein nanoemulsions of required droplet size ranges are produced. In low energy methods such as emulsion inversion point, the composition is changed by dilution at room temperature [129]; in the phase inversion temperature method, temperature is increased above the phase transition of the surfactant mixture and then cooled down to ambient temperature, resulting in the transformation of w/o to an o/w, or vice versa. The hydrophilic-lipophilic balance value of the surface-active agent is critical for the preparation of nanoemulsion by the phase inversion method. Though the emulsification process is spontaneous, the coalescence rate and instability of emulsion are the main issues related to this technique. In the phase inversion composition technique, the composition of the phases is altered by adding a hydrophilic-lipophilic balance transforming agent, leading to the formation of nanoemulsion [130]. The main drawbacks of these techniques are complexity, extended preparation time, the large tank required by the cooling process, and expense. Dilution of dispersed phase carried out at constant temperature leads to spontaneous nano emulsification without any phase inversion [131].

#### 5.3.2. In Vitro Characterization Techniques for Nanoemulsion

The first step in the vitro characterization technique is visual inspection or light transmittance technique to check the clarity to examine potential physical instability issues during processing and storage. An ideal pH and osmolarity are compulsory for ophthalmic formulation to avoid tissue irritation, retain corneal integrity and maintain clinical performance. The antimicrobial efficacy of the nanoemulsion is evaluated by incubating it with probable pathogens at a specific concentration; viable microorganisms are tested by culturing them in suitable media, as per the protocol and procedure mentioned in ISO 11930 and USP Chapter <51> [131]. A particle size distribution study should be conducted to evaluate the physical stability of the formulation stored under different storage conditions, as per the ICH guidelines. In vitro and ex vivo tests are conducted to find the release pattern of the drug from the encapsulated nanodroplets and to assess the permeation of actives across the ocular tissues. Pharmacokinetic evaluation can be conducted to find the ocular bioavailability and clearance of the drug from the targeted site in the ocular tissues [132]. Ocular sensitivity test usually based on Draize technique is done to determine the potential of a nanoemulsion or ingredient to cause eye irritation when administered by the patient [133]. Sterilization of nanoemulsion can be carried out by either moist heat or membrane filtration under aseptic conditions. Different types of characterization techniques typically utilized for the evaluation of nanoemulsion are summarized in Table 5.

#### 5.3.3. Functional Role of Nanoemulsion in Ocular Drug Delivery

The low viscosity of the nanoemulsions posed a novel obstacle to the formulation scientist to prolong the contact time with the ocular epithelial surface. Nanocarrier formulation can be converted to in situ forming gels based on the type of polymer and change in pH [144], temperature [145], and electrolyte triggered [146] in the eye. This would increase the viscosity of the preparation; hence leading to an increase in contact time, sustained release, an increase in intraocular penetration, and subsequently an improvement of ocular bioavailability. Nanoemulsions have many advantages over conventional emulsions but suffer from certain limitations, such as ocular sensitivity reactions contributed by high surfactant content, and cloudy vision because of the increased viscosity of the formulation. Based on thermodynamic principles, nanoemulsions are inherently unstable systems that may undergo time-dependent physical instability problems, such as flocculation, creaming, coalescence, phase separation, and Oswald ripening [147].

Various studies indicated that the chemical properties of the excipients, such as lipid, surfactants and polymers, play a key role in the stability as well as the sustained release profile of the nanoemulsions. Blood–retinal barriers can be effectively permeated by nanoemulsions as shown by the extended-release pattern of lutein up to 12 h, detected with fluorescence in the retina from penetratin-modified lutein nanoemulsions dispersed within in situ gel [148]. The electroretinography study found that the treatment group’s visual function was improved when compared to the control group and that the effect of penetratin-modified lutein nanoemulsions in situ gel was the greatest.

**Figure 5:**
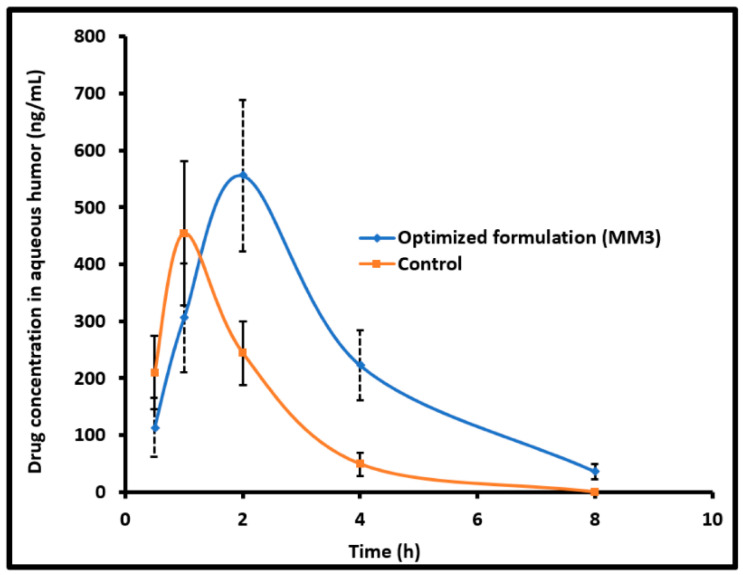
A comparative evaluation of moxifloxacin level in the aqueous humor of rabbits from optimized nanoemulsion and control (commercial moxifloxacin eye drops) with similar dose (adapted from [35], published by MDPI, 2019).

Using the pseudo ternary phase diagram and aqueous titration approach, we were able to successfully encapsulate moxifloxacin in nanoemulsions made from four-component combinations of oil (ethyl oleate), surfactant (Tween 80), cosurfactant (Soluphor P), and water [35]. Ex vivo permeation studies conducted with a Franz diffusion cell using rabbit corneal membrane indicated comparable corneal flux value (32.01 μg/cm^2^/h versus ~31.53 μg/cm^2^/h) for both optimized formulation (MM3) and control, respectively. Ocular tolerance of MM3 indicated good tolerance and storage in a refrigerator for 3 months indicated good physical stability. High aqueous humor moxifloxacin level (*C*max; 555.73 ± 133.34 ng/mL) and decreased *T*max value (2 h) exhibited by MM3 propose a decreased dosing frequency, enhanced therapeutic efficacy and, hence, an improved patient compliance compared to control (commercial eye drops). The aqueous humor *AUC*0–8 h of MM3 (1859.76 ± 424.51 ng·h/mL) was ~2 fold higher (*p* < 0.0005) than the control, thus, demonstrating a major improvement in ocular bioavailability (Figure 5).

Bacterial infections such as ocular keratitis can cause visual impairment; fluoroquinolone derivatives have been recommended as the drug of choice by the US FDA. An investigation was conducted to develop a nanoemulsion loaded with ciprofloxacin to facilitate ocular drug penetration [149]. Ciprofloxacin-loaded nanoemulsion formulations were created using a hot homogenization approach followed by ultrasonication, using oleic acid as the lipid phase, Labrafac^®^ as the lipid phase, and Tween^®^ 80 and Poloxamer 188 as surfactants. Selected drug loaded-NE formulation demonstrated nanosized, uniformly distributed globules indicated by polydispersity index, and zeta potential, respectively. In vitro drug release and ex vivo, trans-corneal diffusion investigations demonstrated controlled release, as well as a 2.1-fold increase in penetrability, respectively, in comparison with marked ciprofloxacin eye drops. Moist heat sterilized nanoemulsion formulation was found to be stable at refrigerated and room temperature for one month. The investigation disclosed that nanoemulsion could provide an efficient ocular delivery carrier for ciprofloxacin and could enhance therapeutic outcomes in bacterial keratitis.

It was reported that positively charged nanoemulsion can be considered as a feasible ocular delivery vehicle in the prevention and probable treatment of ocular neovascular diseases [150]. The therapeutic potential of antisense oligonucleotide (ODN17) encapsulated in cationic nanoemulsion for targeting at VEGF-R2 to decrease neovascularization was studied in mouse models. A marked corneal neovascularization inhibition effect was recorded in the groups applied with ODN17-loaded nanoemulsion administered through both topical and subconjunctival routes. A summary of recently published articles on nanoemulsions-based formulation targeted for various ocular diseases are tabulated (Table 6). The Novasorb^®^ technology platform is utilized to deliver cationic nanoemulsion to negatively charged corneal and conjunctival cells lining the ocular surface at a physiological pH [151]. Furthermore, the high surface area offered by the nanoemulsion droplets creates high contact area with the ocular surface cells, thereby enabling enhanced ocular bioavailability.

### 5.4. Liposomes

Because of the amphiphilic characteristic of the corneal membrane, versatile nanocarriers such as liposomes can act as an efficient and safe ocular transporting agent for various bioactive. Liposomes have spherical vesicular structures that allow polar medications to be loaded into the aqueous core and lipophilic pharmaceuticals to be intercalated into the phospholipid bilayer [36]. Due to many beneficial properties, such as biocompatibility, biodegradability, nano-size, potential, stability, residence time, ability to encapsulate hydrophilic and hydrophobic drugs, internalization and distribution of the drug, liposome is considered as an ideal drug delivery vehicle in the field of ophthalmology [156].

Liposomes can adhere to the corneal cell surface, which increases the ocular residence time, as well as permeation of poorly absorbed drugs. Intracellular delivery of liposomes can be explained by four different mechanisms, namely, adsorption facilitating passive diffusion or transport; endocytosis resulting internalization into endosomes, degradation in lysosomes and release of drug to the cytoplasm [157]; fusion with the lipid bilayer of liposome and lipoidal cell membrane leads to direct delivery to the cytoplasm; lipid exchange due to likeness between phospholipids constituting cell membrane and liposomal lipids, causing destabilization of liposomes and release of actives [158]. Furthermore, liposomes can alter pharmacokinetics, improve clinical efficacy, and decrease toxicity, typically observed with high doses. Various studies indicated that ocular drug absorption was significantly increased when encapsulated in these biocompatible lipid vesicles after topical administration [159]. Liposomes frequently show physical instabilities because of drug leakage, susceptibility for phagocytosis, aggregation, and partitioning to the solvent that may hinder ocular transport. Although there can be suffering from long-term stability due to hydrolysis in solution form or oxidation of unsaturated lipid components, the relevance of liposomes in ocular drug delivery continues because of a simple method of formulation and diverse physical properties. Continuing and completed clinical trials of a liposomal formulation designed for ocular delivery are summarized in Table 7.

#### 5.4.1. Preparation Methods

Based on the size and number of bilayers, liposomes can be classified broadly into multilamellar vesicles and unilamellar vesicles. Unilamellar vesicles are further classified into small unilamellar vesicles and large unilamellar vesicles. Almost all liposome preparation methods generally involve the following stages, such as extraction of lipids from organic solvent and dispersing them in aqueous solvent or buffer, purification of lipids in the formed liposomes, and analyzing the final product [160]. The drug is encapsulated through passive loading during liposome formation or actively after liposome preparation. The mechanical dispersion method, solvent dispersion method, and detergent or nonencapsulated material removal are the major passive loading techniques. The various types of mechanical dispersion methods are sonication, French pressure cell: extrusion, freeze-thawed liposomes, film hydration, micro-emulsification, membrane extrusion, and dried reconstituted vesicles [161]. Sonication is the most frequently used technique for the preparation of small unilamellar vesicles, while multilamellar vesicles are sonicated either using a bath-type sonicator or probe sonicator under a passive atmosphere. The mechanism of French pressure cells involves the extrusion of multilamellar vesicles through a small orifice for producing unilamellar or oligolamellar vesicles (25–75 nm). Rapid freezing and slow thawing of small unilamellar vesicles carried out in the freeze-thawing technique lead to the creation of unilamellar vesicles. In the case of solvent dispersion methods such as ether injection and ethanol injection techniques, lipid dissolved in organic solvent or ethanol is injected into an aqueous solvent or buffer solution containing materials to be encapsulated under reduced pressure [162]. The main disadvantages of these techniques are that the formed vesicle is heterogenous and has chances of inactivation of various bioactive molecules. The reversed-phase evaporation method based on the creation of inverted micelles can entrap a high percentage of water-soluble and amphiphilic molecules. Liposome vesicles are isolated in detergent or non-encapsulated material removal methods based on the principles of dialysis, absorption, gel permeation chromatography, and dilution.

#### 5.4.2. In Vitro Characterization Techniques for Liposomes

Suitable in vitro characterization techniques should be performed for the prepared liposome to evaluate their reproducibility and to ensure the specificity of their desired function [163]. The key features of liposomes are size and size distribution, PDI, entrapment efficiency, surface potential, chemical constitution, lamellarity, morphology, and stability. Size and size distribution is one of the most determinant characteristics of liposomes that provides information about the physical stability, probability of immunity reactions inside the body, quality of liposomes, and batch-to-batch consistency. Electron microscopy methods such as cryo transmission electron microscopy, freeze-fracture transmission electron microscopy, fluorescence microscopy and atomic force microscopy can be used for the visualization and measurement of vesicle size, lamellarity, and morphology [164]. Field flow fractionation methods such as sedimentation, flow, thermal and electrical could be utilized to assess the size distribution and relative molecular mass of liposomes. The important advantage of these techniques is the prevention of degradation of samples and the ability to analyze the size ranges from 1 nm to 1000 nm. The dynamic light-scattering method, also called photon correlation spectroscopy, is extensively employed for the estimation of the size distribution of liposomes. Since this method is rapid, precise, and easy to operate, it could be utilized for the routine measurement of the size distribution of liposomes. The laser light-based nanoparticle tracking analysis method focuses on determining the size, size distribution, and concentration of monodisperse as well as polydisperse liposome suspensions. Liposome size distribution and size inhomogeneity could be determined with the help of flow cytometry [164]. Size exclusion chromatography and high-performance size exclusion chromatography could be employed for the separation of liposomes according to their size besides measurement of size and physical stability. Other reported methods used for the measurement of liposome size are the scanning ion occlusion sensing method and centrifugal sedimentation methods. The physicochemical properties and phase transition of liposomes could be analyzed with a thermal-based technique such as differential scanning calorimetry. The surface charge or zeta potential of the liposomes in the dispersion is routinely obtained by light scattering method. Laser doppler velocimetry is also used for the rapid determination of liposomal surface charge and potential dependent adsorption and for binding to the surface of the liposomes [165]. Encapsulation efficiency of liposomes can be determined based on the separation of unencapsulated drugs from the liposome suspension using the mini-column centrifugation technique. Furthermore, it can also be estimated by the destruction of the lipid layer; the released drug can be subsequently quantified using conventional spectroscopy techniques. Spectrophotometric techniques and enzymatic assay methods are used for the quantification of individual components of liposomes. The lamellarity of the liposomes influences the entrapment efficiency, release kinetics, and pharmacokinetics of the enclosed actives and therapeutic applications. The 31P nuclear magnetic resonance technique, chemical reagent method, and small-angle X-ray method could be used to determine the lamellarity of the liposomes in dispersion [166].

#### 5.4.3. Strategies to Improve Ocular Liposomal Drug Delivery

The surface charge, lipid composition, physicochemical nature of the encapsulated agents, and the interplay between the drug and the vesicles are the factors that determine the effectiveness of liposomes in drug delivery [167]. Various bioadhesives and penetration enhancing polymers are evaluated for ocular drug delivery targeting diseases affecting the anterior part of the eye. Optimization of ocular drug delivery systems based on positively charged mucoadhesive polymers would potentially entrap the particles in the negatively charged mucin layer due to electrostatic interaction. Thus, it is predicted that the association of cationic multilamellar liposomes with the corneal surface is stronger compared to other types of liposomes [168]. Utilization of mucoadhesive hydrophilic, biocompatible polymers such as chitosan and PEG are preferred for extending precorneal residence time since they have added benefits of protein shielding effect and penetration enhancing abilities [169]. Thus, retention of nanoparticles within the cul-de-sac after administration is essential for sustained drug release effect and prolonged therapeutic effect. To avoid potential non-specific interaction with non-corneal surfaces, the liposome is coated with mucoadhesive polymers [170]. To improve the targeted corneal attachment, monoclonal antibodies linked to antiviral-loaded liposomes have been developed [171]. Recombinant human IgG1 monoclonal antibody, Adalimumab (Humira^®^) was approved by FDA for the treatment of non-infectious intermediate, posterior, and panuveitis [172]. In vitro studies showed good corneal interaction with immunoliposomes however poor penetration into the stroma layer limits its performance in ex vivo experiments. Though positively charged liposomes significantly improved the ocular residence time, liposomes prepared with neutral or negatively charged phospholipids such as stearylamine and CTAB showed appreciable cytotoxicity [173]. The increased ocular retention was due to the molecular association of cationic lipids with polyanionic corneal and conjunctival mucoglycoprotein mainly dictated by the charge density and cohesive strength of the lipid bilayer. Based on the same approach, researchers also probed lectin conjugated liposomes, and cationic lipid analogs [158].

In vitro corneal permeation and in vivo ocular absorption in rabbits reported that liposomal surface charge is a crucial factor that impacts the performance of ocular drug delivery systems. Cationic liposomes entrapped with acyclovir demonstrated higher drug entrapment efficiencies, rapid drug delivery rates, the enhanced penetration rate in comparison to negatively charged and neutral acyclovir encapsulated liposomes [174]. The prominent role of cationic liposomes in ocular delivery was further endorsed by increased AUC (1.5 fold) and delayed Tmax (2 h), demonstrated by ibuprofen-loaded cationic liposomes compared to ibuprofen eye drops [175]. Intravitreal administration of liposomal drug delivery system with or without PEG coating using ex vivo animal model using fresh porcine eyes has been investigated [176]. It was demonstrated that positively charged liposomes coated with PEG absorbed quickly to the retina (<1 h) and remained there for a period of 24 h. Further, the pharmacokinetic evaluation indicated a 45-fold enhancement in the vitreous half-life of fluorophore calcein encapsulated in liposomes in comparison to the drug solution. It is assumed that liposomes with positive charge diffuse slower in the vitreous body mainly due to the presence of constituents such as hyaluronic acid and heparan sulfate [177]. In contrast, the liposomal surface charge did not prove to improve the topical ocular besifloxacin delivery via iontophoretic treatment [178]. Both commercial and liposome formulations demonstrated an identical increase in permeability rate consequent to the electrical charge application. 

Chitosan-coated liposomes showed an increased precorneal residence time and decreased drug metabolism at the epithelial surface of the corneum. Employing liposomes alone has limited applicability due to nano-size and surface potential, which leads to low entrapment efficiency, minimum residence time, and limited penetration. A comparative investigation was conducted between chitosan-coated liposomes and conventional liposomes encapsulated with triamcinolone acetonide based on drug entrapment and release properties. The film hydration method was used to prepare liposomes that were subsequently dispersed in chitosan under stirring at room temperature to provide a coating. Chitosan-coated liposomes demonstrated enhanced drug loading efficiency (74%), a high positive zeta potential (+41.1 mV), and prolonged residence time [179]. The maximum drug release rate recorded for drug encapsulated plain liposomes was 93% at the 8th hour while drug release rates from 0.2% and 0.3%s chitosan-coated liposomes were 83% and 73%, respectively. In addition, a large quantity of triamcinolone was estimated in the ocular tissues after two weeks of treatment with choroidal neovascularization rat models. The results here signify the immense potential of this novel nanocarrier to develop as a promising application in the ocular drug delivery system for treating particularly posterior segment diseases.

The antimicrobial efficacy of chitosan-coated liposomes embedded with ciprofloxacin was also investigated [180]. It was suggested that pseudoelastic properties of chitosomes are responsible for extended residence time and long-term stability of tear film, besides ionic interactions between oppositely charged polymers. In addition to the electrostatic attraction between cationic chitosan and anionic mucin, hydrogen bonding involving chitosan with the hydrophilic ocular surface prolongs precorneal residence time. Furthermore, in vitro release studies reported a slower release rate from chitosomes due to the extra diffusional barrier imparted by the coated layer of the chitosomes. Ex vivo diffusion studies employing isolated rabbit corneal membrane showed 1.7-fold enhanced permeation with coated liposomes compared to the free drug due to penetration enhancement properties of chitosan. Ciprofloxacin encapsulated chitosomes showed better antibacterial effect than commercial drug solution when tested with pathogenic microbial strains of *Pseudomonas aeruginosa* and *Staphylococcus* aureus for 24 h. It was proposed the antibacterial activity is contributed by the electrostatic attraction between the cationic chitosan and negatively charged microbial cell membranes. Though this investigation revealed that medium molecular weight chitosan is more effective for liposome coating, results from other research indicate the benefits of using aqueous-soluble chitosan of low molecular weight as a suitable coating polymer.

Functionalization of liposomes can often decrease the particle aggregation tendency of the liposomes and, thereby, improve the physical stability. Surface modification of diclofenac-loaded liposomes with hydrophilic PVA and corresponding derivatives carrying a hydrophobic center was found to improve the stability. Enhanced physical stability and decreased particle agglomeration were noticed in addition to strong mucoadhesion characteristics because of increased chain flexibility and better dispersion capability. The retinal transport of diclofenac was greater with PVA-coated liposomes in comparison to non-liposomal formulation [181].

#### 5.4.4. Role of Liposomes in Ocular Drug Delivery

Dry eye disease is typically characterized by the absence of adequate homeostasis of the tear film resulting in inflammation, soreness, and visual impairment. Lactoferrin, a glycoprotein with anti-inflammatory, antibacterial, antiviral, antifungal, and immunomodulatory properties has been studied for different ophthalmic conditions. Lactoferrin loaded into liposomes coated with hyaluronic acid was prepared by the conventional lipid hydration method followed by high-pressure homogenization [182]. The nanosized, homogenous particle size distribution, positive zeta potential, and good encapsulation effectiveness of the prepared liposomes were observed. Developed liposome formulation showed significant ability to reverse the symptoms of the dry eye without inducing any ocular toxicity.

**Figure 6:**
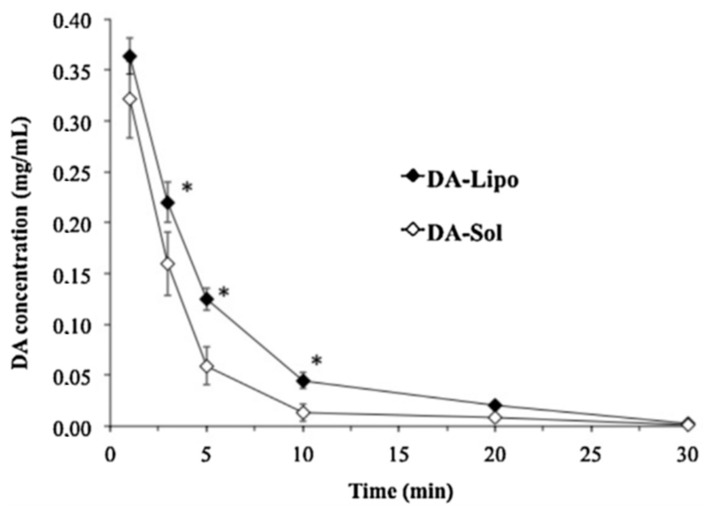
Lachrymal dexamethasone concentration-time profiles upon topical instillation of the dexamethasone-liposome formulation in comparison to dexamethasone solution with similar dose. * Significantly different from DA-Sol (*p* < 0.05, *t*-test). (adapted with permission from [183], published by Elsevier, 2015).

In vitro and in vivo studies performed utilizing controlled release liposomal formulation comprising distamycin A in rabbits displayed enhanced drug concentration compared to IC_50_ values reported for distamycin A-solution (DA-Sol) against Herpes simplex virus (HSV) ocular infections without any indications for corneal penetration [183]. The ocular bioavailability study disclosed that, soon after installation, the drug concentration in the lachrymal fluid was the same for both distamycin A- liposome (C_min_ = 0.364 ± 0.018) and DA-Sol (321 ± 0.038 mg/mL). The drug concentration in the lachrymal fluid was substantially more at 3, 5, and 10 min (*p* < 0.05) for distamycin A-liposome formulations when compared to the reference formulation. However, after 30 min, the concentration of distamycin A was found to be more than IC_50_ values reported for HSV1 from both preparations, but for HSV2 the results indicating IC_50_ and distamycin A concentration were identical only for distamycin A-liposome. These results were demonstrative of a slow clearance of distamycin A after topical applications of the liposome preparation. The pharmacokinetic analysis reported the half-life of distamycin A for DA-Sol and distamycin A- liposome in lachrymal fluid as 1.82 and 2.75 min, respectively. The AUC values conformed to higher ocular bioavailability (1.73 fold) from distamycin A- liposome formulation compared to DA-Sol. A large quantity of distamycin A was present in the ocular structures 30 min following application with DA-Sol (1.579 ± 0.087) and distamycin A- liposome formulations (2.028 ± 0.063 ng/mg), confirming the strong binding of the liposomal and the corneal structures. It was proposed by the investigators that small nanoparticles may remain within the corneal and/or the scleral layers promoting the corneal permeation of the drugs while macro-sized nanoparticles might be remained beneath the eyelids or in the inner canthus, thereby extending the contact time of the drug. The suggested hypotheses along with cytocompatibility of distamycin A- liposome may elaborate the extension of the precorneal residence time of distamycin A and the two-fold increment of ocular bioavailability of distamycin A- liposome formulation (Figure 6).

Employing a mechanically strong mucoadhesive polymer is an effective technique to increase the ocular residence time and, thereby, enhance ocular therapeutic efficacy. Liposomes coated with natural macromolecular fibrous protein, silk fibroin for extended ocular drug transport has been described [184]. Liposomes loaded with ibuprofen were coated with the regenerated silk fibroins having varying dissolving times. Though the initial rapid release was noticed in the ibuprofen solution, such a drug release pattern was drastically decreased in the silk fibroin-coated liposomes. Silk fibroin-coated liposomes displayed sustained drug release behavior compared to uncoated liposomes and, as expected, the release rates slow down as the concentration of silk fibroin concentration was increased. Due to an increase in silk fibroin concentration, stronger interaction between protein structures can take place resulting in a high degree of β-sheet structures. This would increase the coating layer thickness and subsequently prevent the burst drug release from the coated liposomes. In addition, the time to dissolve silk fibroin could affect the compactness of the silk fibroin coating due to the variation in the amino acid sequences of the protein. A linear increase in drug diffusion from silk fibroin-coated liposomes was observed after lag time besides achievement of sustained-release behavior due to silk fibroin coating. The fluorescence intensity generated from Nile red was higher in silk fibroin-coated liposomes compared to drug-loaded conventional liposomes confirming a quick and steady entry to the corneal epithelial cells up to 2 h by silk fibroin coated liposomes. The survival rate or cell viability of the transfected cells was estimated based on the mitochondrial conversion of NDPH dependent oxidoreductase enzyme using tetrazolium salt, 3-(4,5-dimethylthiazol-2-yl)-2,5-diphenyl tetrazolium bromide assay proving that silk fibroin and silk fibroin coated liposomes did not cause any detectable cytotoxicity. These favorable characteristics of silk fibroin-coated liposomes recommend them as a potential and feasible choice for an efficient ocular drug delivery system.

HSV keratitis has been recognized as the frequent cause of corneal blindness or severe mononuclear visual impairment, affecting nearly 1.5 million people worldwide [185]. Drug-loaded liposome formulation can be dispersed as patient-friendly ophthalmic drops for the therapy of different ocular disorders. In situ gels have been widely investigated as ocular drug delivery systems to improve bioavailability and therapeutic efficacy [186]. Transparent in situ stimuli-responsive gel formulation is suitable for ocular delivery as it can be administered as droppable dosage form while avoiding potential visual disturbances. Furthermore, it has better mucoadhesion, ocular tolerability, and sustained release profile compared to the conventional formulation due to extended contact time with the ocular surface [25]. Thus, liposomal formulation dispersed in in situ gel matrix can control the drug release without causing increased drug concentration at the target tissue site and ensuing ocular toxicity. Methazolamide liposomal in situ gel has been prepared with a conventional lipid film hydration method using lecithin and cholesterol. The methazolamide liposomal gel exhibited sustained drug release and a major reduction (*p* < 0.05) in intraocular pressure compared to pure drug solution [187].

A targeted drug delivery system is beneficial since it can avoid typical undesirable effects and potential drug interactions. Liposomal fluconazole formulation (2 mg/mL) administered through topical route three times per day in one month was shown to be highly effective in patients suffering from an ocular infection caused by Candida keratitis [188]. The liposomal formulation was encapsulated with moxifloxacin and dexamethasone-loaded nanostructured lipid nanoparticles and later mixed with collagen/gelatin/alginate for extended ocular application [189]. The prepared non-toxic ocular formulation showed nanoparticle size, negative zeta potential high encapsulation capability, and drug loading. In addition, minimum effective concentration for corneal keratitis was achieved within an hour; the drug release was sustained for a period of at least 12 h. Inhibition of microbial growth and improvement of wound healing was observed after animal study.

#### 5.4.5. Functionalization Strategies of Liposomes

Stealth liposomes or long-circulating liposomes are surfaces coated with inert, biocompatible hydrophilic polymers such as PEG to avoid identification by opsonins and subsequent clearance via mononuclear phagocyte system [190]. The covalent links of PEG chains present on the surface of liposomes enhance their entrapment and accumulation at the target cells. The PEG coating is removed at the acidic pH of the inflammation site in pathological conditions [191]. PEGylated liposomes are considered to be a safe and effective approach for ocular gene transfer [192]. The PEG has many benefits, such as non-ionic, high solubility both in aqueous and non-aqueous solvents, and biocompatibility [193]. Nevertheless, PEGylation needs a large quantity of stabilizer and cholesterol to prevent undesirable interactions between PEG chains, which can lead to agglomeration. However, it was reported that frequent administration of PEGylated nanocarriers leads to antibody-mediated accelerated blood clearance phenomena, which reduces the safety and efficacy aspect of encapsulated drugs [194]. In addition, there is a chance of hypersensitive reaction, known as complement activation-related pseudoallergy, which can adversely impact the clinical translation of PEGylated products. Therefore, nanotherapeutics should be screened for immunogenic reaction tests before initiating clinical studies. Though PEG continues to remain as standard coating material, several alternate hydrophilic polymers, polyoxazolines, zwitterionic hydrophobic polymers have been evaluated to circumvent the limitations of PEG [195].

To achieve site-specific delivery of the drug, targeting ligands such as immunoglobulins and their fragments are frequently attached to the liposomal surface for targeted delivery without affecting their integrity. To deliver non-viral vectors to endothelial surface receptors, immunoliposomes have been developed [196]. Non-viral systems such as cell-penetrating peptides can be considered as a feasible option to overcome the low membrane permeability of these charged macromolecules [197]. To facilitate trans-corneal drug administration and lengthen ocular surface residence, cell-penetrating peptide TAT-functionalized, flurbiprofen-loaded liposomes were recently developed [198]. The corneal permeation-enhancing properties of TAT-functionalized liposomes (TAT-Lip) were demonstrated in vitro using the HCE-T cell sphere model and in vivo using aqueous humor pharmacokinetics. The electrostatic interaction between cell-penetrating peptide TAT-liposomes cell membrane resulted in the partial opening of tight junctions and, thereby, cellular internalization. The therapeutic efficacy of TAT-flurbiprofen-liposomes was increased by marked suppression of inflammatory mediators, namely, PGE_2_, IL-6, and TNF-α secretion in lachrymal fluid in tears and aqueous humor in a rabbit conjunctivitis model. To avoid the loss of drugs by enzymatic degradation during endocytosis, fusogenic lipids or peptides are typically used. This will destabilize the membrane after conformational activation and subsequently deliver the drugs directly to the cytoplasm at the low pH of endosomes. Thus, pH-sensitive liposomes have the potential ability for high cytoplasmic drug delivery. Many investigations are currently being conducted to evaluate the targeting ability of pH and temperature-sensitive liposomes and stealth liposomes [199].

Due to the beneficial property of overcoming the various physiological barriers of the eye, lipid nanoparticles are considered as a novel formulation strategy for the treatment of posterior segment eye diseases. Liposomal formulation loaded with triamcinolone acetonide (0.1–0.2%) was found to be as effective as a combination therapy for the prevention of macular edema associated with laser-assisted cataract surgery [200]. Clinical trial evaluation of liposome-based ophthalmic formulation comprised of dexamethasone sodium phosphate (ProDex^®^) displayed better therapeutic efficacy to retinal occlusion as demonstrated by a reduction in retinal central subfield thickness and enhancement of visual acuity [201].

Though liposomes are used to decrease the systemic toxicity generated by the loaded agent, they can cause toxicity to normal tissues and, hence, can initiate an immunogenic response. Cationic liposomes are extensively evaluated for gene delivery, are known to trigger toxicity in macrophages and to modify the secretion of prominent immunomodulators [202]. Despite having many benefits of using liposomes in drug delivery, these limitations must be addressed before proceeding to clinical translation.

## 6. Future Perspectives

SLNs combine the beneficial properties of both liposomes and polymer carriers besides having the ability to encapsulate hydrophilic, as well as lipophilic, drugs. SLNs carry significant potential of targeted and constant drug delivery and the potential capacity to penetrate all ocular tissues, including the posterior chamber, which has recently drawn the attention of many researchers worldwide. Recent investigations revealed that drug-loaded SLNs can penetrate phagocytic cells and cross bacterial membrane barriers, thus, opening new horizons for the treatment of ocular infections and defining a strategy to overcome the challenges of microbial resistance. Several SLN based patents have already been filed and more functionalized SLNs based drug delivery systems would rapidly emerge soon. With the current progression and efforts being undertaken in ocular research, it is anticipated to result in high ocular residence time, to restrict non-specific tissue accumulation and to deliver therapeutic drug concentration into desired ocular tissue sites besides replacing invasive modes of drug administration, such as periocular and intravitreal injection. The application of quality-by-design-based approaches could ease and optimize the formulation development of nanocarrier systems, particularly during the early preformulation stage [203]. Rapid progress in lipid nanoparticle research resulted in the approval and commercialization of ophthalmic drug products such as Restasis^®^, Ikervis^®^, or Cequa^®^. NLCs are widely probed as carriers for the transport of drugs to the eye due to their improved drug loading and permeation properties besides their sufficient safety profile. Nanoemulsions are a comparatively most effective delivery vehicle to increase the solubility, bioavailability, and functionality of hydrophobic compounds, which further affirm their practical relevance in ocular drug delivery systems. The utilization of nanoemulsions continues to hold challenges of thermodynamic instability that need to be addressed for successful scale-up for the production process, patient safety, and acceptance. Targeting posterior segmental diseases using nanoemulsions having high drug loading capacity and sustained/controlled release profile by non-invasive route would be the future direction of research. After the approval of several liposome-based drug products for clinical use, tremendous progress has been taking place concerning liposome lipid-drug conjugates for enhanced trans-corneal permeation and targeting. The development of biocompatible and biodegradable polymers, delivery of biotechnology, and tissue engineering products are urgently required now for immediate advancement in the field of ophthalmology.

## 7. Conclusions

Targeting drugs to the posterior chamber of the eye through the topical route is still a difficult task because of formulation constraints and the complex anatomical, physiological, and efflux barriers that exist in ocular tissues. Lipid nanocarriers demonstrated excellent ocular permeation characteristics and penetration-enhancing capabilities besides displaying high drug loading and entrapment efficiencies. Lipid nanocarriers will be of considerable interest to researchers who aspire to design and develop ophthalmic drug products with improved efficacy, safety, and acceptability. Drug transporter proteins such as P-gp and multidrug efflux pumps are found in the eye; therefore, lipid nanocarriers could be utilized to target these transporters to promote ocular bioavailability with reduced toxicity. Coordinated efforts between academia, industry, and regulatory authorities are vital to facilitate the potential of these nanoparticles while addressing issues of safety and efficacy.

## Figures and Tables

**Table 1:** Structural components of solid-lipid nanoparticles.

Type	Examples	Reference
Lipids	Beeswax, Behenic acid, Carnauba wax, Cetyl palmitate, Glyceryl behenate (Compritol 888 ATO), Glyceryl caprate, Glyceryl monooleate, Glyceryl monostearate (Imwitor 900), Glyceryl palmitostearate (Precirol ATO 5), Labrafil M1944, Miglyol 812, Monostearin, Oleic acid, Palmitic acid, Paraffin, Polyethylene glycol monostearate, Stearic acid, Trilaurin, Trimyristin (Dynasan 114), Tripalmitin (Dynasan 116), Tri-stearin (Dynasan 118), Witepsol, etc.	[59,60]
Emulsifiers	Butanol, Butyric acid, Cetylpyridinium chloride, Cremophor EL, Eumulgin SML 20, Lecithin, Poloxamer 188, Poloxamer 407, Polysorbate 20, 60, and 80, Polyvinyl alcohol, Sodium cholate, Sodium deoxycholate, Sodium dodecyl sulphate, Sodium glycocholate, Sodium oleate, Taurodeoxycholic acid sodium, Tyloxapol, etc.	[61]

**Table 2:** List depicting various solid lipid nanoparticles preparation methods, procedures, advantages, and limitations.

Method	Procedure	Mechanism	Advantages	Limitations	Reference
High-pressure homogenization Hot homogenization technique	The temperature of solid lipids is kept above their melting point. At this point, actives can be added. At the same temperature, the molten mixture was added to an aqueous solution with a stabilizing agent. Solution or dispersion subjected to homogenization under high pressure (400–800 bar) resulting in high velocity (27.78 m/s) stream subjected to intensive turbulent physical forces	Submicron particle size is generated by forming high shear forces, cavitation forces, current of eddies, and pressure distortions in the mixture	Useful for thermostable drug, efficient dispersion technique to obtain nano-size range particles (50 nm–400 nm), low risk of product contamination, allows aseptic production of nanoparticles, and easy to scale-up	High polydispersity, the chance of metal contamination, unsuitable for the thermolabile drug due to heat generation during the process, expensive equipment. Coexistence of supercooled melts, various colloidal structures during lipid crystallization, and partitioning of drug towards the aqueous phase	[65,66]
Cold homogenization technique	A melted mixture of lipid is cooled and milled to coarse dispersion having particle size range (50 μm–100 μm). Subsequently distributed in water containing the emulsifying agent and homogenized at room temperature		Feasible for thermolabile drugs; the coexistence of other colloidal structures is minimum	Prerequisite of micronized drug particles in dispersion before homogenization step	[67]
Microemulsion technique	The microemulsion is formed by dispersing molten lipids with an aqueous solution of surfactant and cosurfactant at the same temperature. Hot microemulsion diluted with an excess quantity of cold water at a ratio of 1:25 to 1:100 resulting in the spontaneous formation of SLN	Negative surface free energy contributed by a large reduction of interfacial tension and large changes in the entropy of mixing	Kinetically stable and is a low energy process	Requires a large amount of surfactant and cosurfactant, and highly diluted preparation requires an additional processing step for product concentration	[68]
Supercritical fluid technique	Gas saturated solution containing lipid material. Supercritical fluid containing lipid material and gas saturated solution under pressure is sprayed through nozzle or atomizer under high pressure	Expansion of solution leads to escape of gas and rapid precipitation nanoparticles	Organic solvent-free process, obtain particles as a dry powder, and wide range of miscibility of lipids in gases	Expensive process and equipment	[64]
Solvent emulsification/ evaporation method	The aqueous phase is combined with lipid material that has been dissolved in an organic solvent. The coarse emulsion is nanosized with a high-speed homogenizer and high shear homogenizer. Evaporation of organic solvent leads to precipitation of nanoparticles	Emulsification of globules followed by evaporation of organic solvent leads to precipitation of nanoparticles	Low energy process, uniform size particles <25 nm, and suitable for thermolabile drugs	The insolubility of lipids in organic solvents, thermodynamically unstable, the presence of residual solvent requires additional drying or ultrafiltration procedure, and toxicological consideration	[69]
Solvent emulsification-diffusion method	Lipid dissolved in organic solvent stirred with a partially miscible aqueous solution containing surfactant. Evaporation of organic solvent carried out by high-speed homogenization followed by high shear homogenization	Spontaneous diffusion of hydrophilic solvents resulting in the creation of interfacial turbulence, following the evaporation of the organic solvent, and nanoparticles precipitate	Low polydispersity with an increase in the concentration of hydrophilic solvents, particle size decreases, suitable for thermosensitive drugs	The insolubility of lipids in organic solvents. Thermodynamically unstable. The presence of residual solvent requires freeze-drying or ultrafiltration techniques. Toxicological issue	[70]
Double emulsion	Water-in-oil (w/o) emulsion containing lipophilic surfactant is dispersed in an aqueous phase with a hydrophilic surfactant to formulate water-in-oil water (w/o/w) multiple emulsions. Nanoparticles are formed by continuous stirring and the evaporation of the solvent	Evaporation of solvent from thermodynamically unstable multiple emulsion leads to solidification of emulsion and lipid crystallization	Suitable for hydrophilic and peptide-based drugs, surface modification of nanoparticles is possible by incorporating hydrophilic polymer	Tends to form large particles and the requirement of multiple steps	[71]
Phase inversion temperature	Holding w/o emulsion prepared above a phase-inversion temperature of non-ionic surfactant with continuous stirring and rapidly cooled below the crystallization temperature of the emulsified phase led to the formation of SLNs	During heating, dehydration of ethoxy groups and increased lipophilicity of surfactants. The system crosses a threshold of zero surfactants happens. Spontaneous curvature and minimum surface tension during cooling, favoring the creation of finely dispersed nanoparticles	The low energy emulsification process, requires only a limited quantity of surfactant, capable to produce uniform size nanoparticles, and economical	Low stability and several temperature cycles may be required	[72]
Membrane contractor	Fine droplets are formed when the melted lipid phase is driven through pores of a membrane that is held above melting temperature. Droplets formed at the outlets are swept into an aqueous medium comprising surfactant flows tangentially to the membrane surface and cooled to room temperature, resulting in the production of SLNs	Emulsification of droplets takes place spontaneously at the interfacial surface of the membrane	Changing the flux through the membrane control the particle size, and feasible scale-up process	Many formulation and process parameters are involved, and the membrane prone to clogging	[73]
Solvent injection	To dissolve lipids and medications, a water-miscible solvent or a water-miscible solvent mixture is utilized. Under continuous mechanical agitation, the organic phase is swiftly injected into the aqueous phase containing surfactant or surfactant combination using a needle	Solvent diffusion from lipid to the aqueous medium. Interfacial cavitation and vibration broke down solvent-lipid droplets to a nano-size and lipid sedimentation	Simplicity, clarity, speed of output, and lack of a complicated instrument	Additional step required for residual solvent removal	[39]

**Table 3:** Examples of solid-lipid nanoparticles in ocular drug delivery and their characteristics.

Therapeutic Category	Lipid Constituent	Surfactant/Charge Modifier	Formulation	Method	Drug	Highlights	References
Antifungal	Precirol ATO 5^®^	Pluronic F68/Stearyl amine	Ophthalmic suspension	Hot emulsification-ultrasonication technique	Natamycin	The selected formulation demonstrated an average particle size of 42 nm, a zeta potential of 26 mV, entrapment efficiency of ~85%, and a prolonged drug release profile of 10 h. Permeability coefficient and steady-state fluxes were 11.59 × 10^−2^ cm h^−1^ and 3.94 mol h^−1^ compared to 7.28 × 10^−2^ cm h^−1^ and 2.48 mol h^−1^ reported for the plain drug, respectively	[88]
Anti-inflammatory	Compritol^®^ 888 ATO (glyceryl behenate) and glyceryl monostearate	Tween^®^ 80	Gels	Film hydration	Triamcinolone acetonide	The trans-corneal permeability of drug-loaded SLNs and drug-loaded SLNs in the gel was 10.2 and 9.3-folds higher when compared to an equivalent dose of drug suspension Drug-loaded SLNs in gel outperformed drug-loaded SLNs and drug suspension in terms of pre-corneal residence time and sustained drug distribution into the anterior and posterior chambers of the eye	[89]
Anti-hypertensive	Glyceryl monostearate and soy lecithin	Tween^®^ 80	Gels	High shear homogenization with sonication	Bimatoprost	Ex vivo trans-corneal permeation of drug-loaded SLNs in gel showed prolonged release (95.43% in 19 h). HET-CAM test confirmed non-irritant nature while histopathological studies indicated non-toxic characteristics of the formulation	[90]
Anti-viral	Stearic acid and tristearin	Poloxamer 188 and sodium taurocholate	Ophthalmic suspension	Solvent-emulsification-evaporation method	Valacyclovir	Ex vivo studies exhibited enhanced drug permeation of SLNs compared to the drug solution. In vivo study confirmed enhanced ocular bioavailability of valacyclovir (AUC_0–12_: 856.47 ± 7.86 μg h/mL) than drug solution (AUC_0–12_: 470.75 ± 8.91 μg h/mL). The non-allergenicity of SLNs was confirmed by histopathology and the Hen’s Egg Test Chorio Allantoic Membrane assay	[91]
Anti-bacterial	Stearic acid	Epikuron 200/sodium taurocholate	Ophthalmic suspension	Hot o/w microemulsion technique	Tobramycin	Application of tobramycin-SLN resulted in deeper penetration to the retina. Demonstrated higher antibiotic concentrations in phagocytic cells compared to the tobramycin reference formulation	[92]

**Table 4:** Selected examples of nanostructured lipid carriers based on ocular formulations and key findings.

Drug	Constituents	Method	Highlights	Reference
Curcumin	Compritol™ 888 ATO and Gelucire^TM^ 50/13 (Solid lipids), Olive oil (Liquid lipid), Vitamin E TPGS (Emulsifier), Poloxamer 188 (Non-ionic Surfactant)	Hot melt emulsification and ultrasonication	Optimized NLC-based on central composite design displayed a uniform distribution (PD1 of 0.17 ± 0.05), particle size (66.8 ± 2 nm), high encapsulation efficiency (96 ± 1.6%), and drug loading of 3.1 ± 21 0.05% *w*/*w*. The flux of the suspension was found to be 0.002 μg/min/cm^2^ while, for the curcumin embedded NLCs, it was 0.005 μg/min/cm^2^. Significant enhancement of curcumin permeation (~2.5 fold) through the rabbit cornea was observed for curcumin encapsulated NLCs compared to the control	[104]
Dexamethasone	Phospholipid (Solid lipid), Soyabean oil (Liquid lipid), Pluronic F127 and F68 (Non-ionic Surfactants)	Hot high-pressure homogenization	Improved precorneal retention time and steady sustained drug release noticed with prepared NLCs. Aqueous humor pharmacokinetics study showed one-fold and three-fold enhancement (AUC_0–12 h_) of NLCs-gel, when compared with NLCs and tobramycin dexamethasone eye drops, respectively	[105]
Flurbiprofen	Stearic acid (Solid lipid), Miglyol^®^ 812 and Castor oil (Liquid lipid), Tween-80 (Non-ionic surfactant)	Hot high-pressure homogenization	The optimum nanoformulation composition is 3.2% *w*/*w* of Tween 80 and 70:30 between stearic acid and liquid lipid. Demonstrated nanosize (228.3 nm), uniform distribution (0.156 PDI), negative zeta potential (−33.3 mV), and high encapsulation (~90%). In vitro study revealed sustained release behavior and no ocular tissue toxicity	[106]
Itraconazole	Tripalmitin (Solid lipid), Capmul MCM (Liquid lipid), Polysorbate 80, and Transcutol^®^ HP (Surfactants)	Hot high-pressure homogenization	Optimized formulation exhibited desirable particle size (86.75 nm), PDI (0.4), and ZP (+25.6 mV), respectively. The whole itraconazole was diffused through dialysis membrane in 2 h from control solution, while sustained drug transport was noticed in itraconazole-NLC and chitosan-coated itraconazole nanoparticles	[107]
Itraconazole	Stearic acid (Solid lipid), Oleic acid (Liquid lipid), (Poloxamer 407 (Non-ionic Surfactant)	High-pressure homogenization	Optimized formulation showed greater entrapment (94.65%), nanosized particles (150.67 nm), and steady drug release (68.67%). Antifungal activity was higher with optimized formulation when compared with commercially marketed products. The in vitro irritation test confirmed that the developed formulation is non-irritant	[108]
Quercetin	Compritol™ 888 ATO and Cremophor EL (Solid lipid), Soy lecithin (Liquid lipid)	Melt-emulsification and ultra-sonication	Optimized quercetin-NLC showed a uniform-sized particle size of 75.54 nm with high encapsulation efficiency (74%). pH and temperature response hydrogel comprised of carboxymethyl chitosan and poloxamer F 127. In vitro study of quercetin-hydrogel showed sustained-release with 80.52% of total quercetin released within 3 days	[109]
Triamcinolone acetonide	Precirol^®^ATO5 (Solid lipid), Squalene^®^ (Liquid lipid), Lutrol^®^F68 (Non-ionic Surfactant)	High-pressure homogenization	Optimization of NLC formulation parameters based on a five-level central composite demonstrated that optimum formulation should be composed of 70% Precirol, 30% squalene, and 2% Lutrol. Triamcinolone concentration (0.025%) was maintained in the partially amorphized lipid matrix with 95% drug loading, good physical stability without any ocular toxicity	[110]

**Table 5:** Various characterization techniques typically utilized for nanoemulsions in ocular therapy.

Technique	Principle	Evaluation Parameters	Reference
Percentage light transmittance	Test samples were placed in the transparent cuvette and checked for transmittance against water as a reference in the colorimeter	Clarity of the nanoemulsion	[134]
Conductivity	The electrode is placed in the sample and the temperature is steadily increased at a rate of 1 °C/min. Nanoemulsion is mixed with a stirrer, and the variation in the conductivity is noted	Identity of the nanoemulsion	[135]
Viscosity	A multipoint viscometer is used to determine the viscosity of nanoemulsions at various angular velocities at a temperature of 34 ± 1 °C. The angular velocity should be raised from 0.5 to 100 rpm and vice-versa having a 6-sec gap between these two speeds	Influences the residence time of the formulation	[136]
Globule size and size distribution	Depending on the intensity and physical features of the dispersed laser light, particle sizes can be determined	Reduction in the globule size improves ocular bioavailability by improved retention in the eyes	[137]
Dilution potential	The prepared nanoemulsions were diluted 10 times with an external phase.	The occurrence of phase separation indicates the stability of the nanoemulsion	[35]
pH and Osmolarity	The pH is measured using a pH meter previously calibrated with standard buffer solutions of pH 4 and pH 7. The osmolarity of the solution is measured by Osmometer. The estimated homeostatic range for tear osmolarity is between 270–310 mOsmol/L	The pH of the ophthalmic formulation should be between 6.5–8.5 to avoid any corneal injury	[138]
In vitro drug release	Conducted in Franz diffusion cell using simulated tear fluid (pH 7.4) as release medium. Between the donor and receptor compartment, an artificial cellophane dialyzing membrane (MW Cut off 12–14 KDa) is placed as a diffusion membrane. The temperature of the receiver fluid is set at 34 °C ± 0.1 °C. The aliquot sample is withdrawn at prespecified time intervals and quantified for drug content	Using various mathematical models, the release data is evaluated to determine the correlation coefficient (r^2^) and release kinetics	[139]
Ex vivo permeation	Optimized formulation or control is kept in the donor chamber and simulated tear fluid (pH 7.4) is placed in the receiver cell (34 °C ± 0.1 °C) of the Franz diffusion cell. An isolated rabbit cornea membrane is sandwiched between the receptor and donor compartment. Samples are withdrawn at various time intervals and analyzed for drug content	The physicochemical properties of the drug, the physiological properties of the membrane, and the permeation pathways available for permeation all influence drug diffusion through the biological membrane. The steady-state flux and the permeability coefficient are computed	[25,140]
Ocular irritation test	In vivo, ocular sensitivity investigations are conducted as per the Draize technique. A single administration of approx. 60 μL is applied in the eyes of albino rabbits (2–3 kg), considered as a treated group, while control groups are treated with normal saline. The sterile formulation is administered twice daily for 21 days	After post-installation, each animal should be checked for ocular sensitivity reactions, such as redness, discharge, conjunctival chemosis, edema, iris, and corneal lesions, and watering of the eyes.	[35]
In vivo pharmacokinetics	Formulation (Test) or control is dropped into the lower cul-de-sac of each eye of an individual group of albino rabbits (2–3 kg), gently close for 2 min to allow for maximum corneal drug contact. Provided local anesthesia at the site and eyelids/eyelashes should be swabbed with povidone (5% *w*/*v*) to follow the normal care to be given before the intra-ocular injection. A 29-gauge syringe needle is used to collect aqueous humor (50 μL) at various time intervals and to assess the drug content	The aqueous humor of rabbit eyes is quantified to evaluate the ocular bioavailability. Onset time, the magnitude of drug action and duration of drug absorption or retention can be evaluated by pharmacokinetic parameters such as T_max_, C_max,_ and AUC	[141]
Pyrogen test	Mix 0.1 mL of the test sample with lysates of amoebocytes of Limulus polyphemus reagent and incubate for 1 h at 37 °C to check for the presence of gel clot. In the direct transfer method, direct inoculation of the test sample in two sample tubes comprising a culture medium i.e., fluid thioglycollate medium, soybean casein digest medium. In the membrane filtration test, the sample is allowed to filter through membrane filters with pores <0.45 μm and diameter 47 mm under vacuum. The membrane is sliced into 2 halves and the individual piece is kept in 2 test tubes comprising soybean casein digest agar to determine total aerobic microbial count and sabouraud dextrose agar to find total combined yeasts and molds	The Limulus lysate test is a more sensitive, specific, reliable cost-effective, and simple method to test endotoxin compared to other methods. Though the procedure is simple, the direct transfer method requires more skill. The membrane filtration method is a more precise method and official in USP	[142]
Stability and shelf-life	Stress stability conditions normally include aging, temperature, centrifugation, and agitation. The stability of the optimized formulation is evaluated on a daily and later weekly for pH, coalescence, droplet size, breaking, flocculation, or precipitation	An increase in temperature causes changes in emulsion parameters such as viscosity, partitioning of emulsifiers, inversion at phase inversion temperature, and crystallization of certain lipids. An increase in gravity, therefore, accelerates the increase in separation of phases	[143]

**Table 6:** An outline of different nanoemulsion-based formulations prepared for various ocular disorders.

Drug	Constituents	Method	Highlights	Conditions	Reference
Brinzolamide	Capryol90 and Triacetin (Oil), Brij 35, Labrasol, Tyloxapol and Cremophor RH40 (Surfactants), Transcutol P (Cosurfactant)	Instantaneous emulsification	Based on the HET-CAM results, only nanoemulsions prepared with Triacetin, Tyloxapol, and Transcutol P (cosurfactant) at 2:1 ratio and Capryol 90, Cremophor RH40, and Transcutol P at 1:1 were classified as non-irritant and slightly irritant, respectively. The penetration of Brinzolamide w/o nanoemulsions through excised bovine cornea was significant compared to the marketed drug suspension	Glaucoma	[133]
Cyclosporine A	Chitosan (Polymer), Oleic acid (Oil), Tween 20 (Surfactant), Transcutol P (Cosurfactant)	Instantaneous emulsification	Tissue distribution studies indicated that chitosan nanoemulsion loaded with cyclosporine A controlled the therapeutic level (≥50–300 ng/g) of cyclosporine A in the cornea and conjunctiva of rabbits up to 24 h. Safety of the formulation was confirmed by Draize test and ocular surface temperature	Dry eye disease, corneal transplant rejection	[152]
Dexamethasone acetate and Polymyxin B sulfate	Eutanol G and Lipoid S 100 (Lipids), Cetylpyridinium chloride (Surfactant), Glycerol	High-pressure homogenization	A novel combinatorial approach utilizing cationic drug and cationic preservative to generate uniform-sized particles (<200 nm) with narrow size distribution. Zeta potential decreased from +9 mV to −11 mV after incubation with mucin. No cytotoxicity was observed after in vitro evaluation and was stable after 180 days	Ocular infection	[153]
Dorzolamide	Isopropyl myristate (Oil), Tween 80 (Surfactant), Cetyl trimethyl bromide (Cosurfactant)	High-speed homogenization followed by ultrasonication	Optimized nanoemulsions exhibited suitable droplet size, zeta potential, polydispersity index, and drug content values. Demonstrated thermodynamic and physical stability. In vitro studies indicated sustained release profile and lowering effect of intraocular pressure in New Zealand rabbits compared to pure drug and marketed eye drops	Glaucoma	[154]
Loteprednol etabonate	Capryol 90 (Oil), Tween 80 (Surfactant), Transcutol P (Cosurfactant)	Spontaneous emulsification	Chosen nanoemulsion demonstrated a low ocular sensitivity index and significantly (*p* < 0.01) elevated C_max_ and AUC_0–10 h_, decreased T_max_, and enhanced bioavailability compared to the marketed formulation	Inflammatory diseases	[155]
Lutein	Lutein, Vitamin E, Egg phospholipids, Medium-chain triglyceride, Ethyl acetate, Gellan gum	High shear mixing, High-pressure homogenization, Rotary evaporation	In vitro release study indicated Fickian diffusion by the nanoemulsion. The nanoemulsion uptake by ARPE-19 cells was confirmed by flow cytometry and confocal microscopy. Inhibitory effect on HUVEC migration confirmed the absence of neovascularization. Shield retinal cells from the injury caused by hydrogen peroxide remove reactive oxygen species in cells.	Age-related macular regeneration	[148]

**Table 7:** Continuing and finished clinical trials of liposome-based formulations evaluated for ocular delivery.

Clinical Trials	Indication	Phase	Enrolment	Identifier
Subconjunctival treatment of liposomal sirolimus as a treatment for dry eye disease. Ocular surface disease index is examined on a scale of 0 to 100, with the highest scores representing greater disability	Dry eye disease	Phase I	52	NCT04115800
Safety and therapeutic effect of liposomal latanoprost in ocular hypertension. Subconjunctival injection of liposomal latanoprost with subjects that have raised intraocular pressure and monitored for pain, inflammation, and toxicity up to 3 months	Ocular hypertension	Phase 1 and 2	6	NCT01987323
Determine the 12-month event-free survival of pediatric patients’ eyes with group D intraocular retinoblastoma treated with systemic chemotherapy, subtenon carboplatin, and local ophthalmic treatment	Intraocular retinoblastoma	Phase 3	30	NCT00072384
Assess the therapeutic potential of a liposomal ozone-based solution (OZODROP^®^) in the preparation of the patient for cataract surgery, by evaluating the reduction of bacterial colonization of the conjunctiva	Ocular infections	Phase 4	200	NCT04087733
TLC399 (ProDex) was studied in participants with macular edema caused by central retinal vein blockage or branch retinal vein occlusion in a randomized, double-masked experiment	Retinal vein occlusion and macula edema	Phase 2	31	NCT03093701
Randomized interventional study wherein Aquoral Forte^®^ was evaluated against Aquoral Lipo^®^ (Cross-linked hyaluronic acid with liposomes and crocin) in dry eye	Dry eye disease is caused by moderate meibomian glands dysfunction	Not applicable	25	NCT03617315
The safety and efficacy of subconjunctival liposomal latanoprost (POLAT-001) vs. latanoprost ophthalmic solution in patients were compared in an open-label, randomized, multi-center, active-controlled parallel study	Ocular hypertension and primary open-angle glaucoma	Phase 2	80	NCT02466399
Randomized interventional trials to evaluate the clinical efficacy of various categories of artificial tears in patients suffering from dry eyes by instilling each category of treatment (0.40% Sodium Hyaluronate (Clinitas Soothe), 0.15% Sodium Hyaluronate (Hyabak), 0.25% Carboxymethylcellulose, electrolyte balanced (Theratears), and Phospholipid liposomal spray (Tears Again)) for a month and examined their tear film and ocular surface after each one	Dry eye	Not applicable	80	NCT02420834

## Data Availability

The data presented in this study is contained within this article.
